# Functional Carbon from Nature: Biomass‐Derived Carbon Materials and the Recent Progress of Their Applications

**DOI:** 10.1002/advs.202205557

**Published:** 2023-03-29

**Authors:** Hongzhe He, Ruoqun Zhang, Pengcheng Zhang, Ping Wang, Ning Chen, Binbin Qian, Lian Zhang, Jianglong Yu, Baiqian Dai

**Affiliations:** ^1^ Department of Chemical & Biological Engineering Monash University Wellington Road Clayton Victoria 3800 Australia; ^2^ Energy & Environment Research Center Monash Suzhou Research Institute Suzhou Industry Park Suzhou 215123 China; ^3^ National Engineering Laboratory for Modern Silk College of Textile and Clothing Engineering Soochow University Suzhou 215123 China; ^4^ College of Chemistry, Chemical Engineering and Materials Science State Key Laboratory of Radiation Medicine and Protection Soochow University Suzhou 215123 China

**Keywords:** biomass‐derived carbon materials, electrocatalysts, environmental applications, functional carbon materials, machine learning methods

## Abstract

Biomass is considered as a promising source to fabricate functional carbon materials for its sustainability, low cost, and high carbon content. Biomass‐derived‐carbon materials (BCMs) have been a thriving research field. Novel structures, diverse synthesis methods, and versatile applications of BCMs have been reported. However, there has been no recent review of the numerous studies of different aspects of BCMs‐related research. Therefore, this paper presents a comprehensive review that summarizes the progress of BCMs related research. Herein, typical types of biomass used to prepare BCMs are introduced. Variable structures of BCMs are summarized as the performance and properties of BCMs are closely related to their structures. Representative synthesis strategies, including both their merits and drawbacks are reviewed comprehensively. Moreover, the influence of synthetic conditions on the structure of as‐prepared carbon products is discussed, providing important information for the rational design of the fabrication process of BCMs. Recent progress in versatile applications of BCMs based on their morphologies and physicochemical properties is reported. Finally, the remaining challenges of BCMs, are highlighted. Overall, this review provides a valuable overview of current knowledge and recent progress of BCMs, and it outlines directions for future research development of BCMs.

## Graphical abstract

Biomass‐derived carbon materials (BCMs) are encountering their flourish moments. This review concludes the current knowledge of BCMs researches and discusses the recent progress of BCMs in various applications. Remaining challenges and future research directions of BCMs are proposed herein.

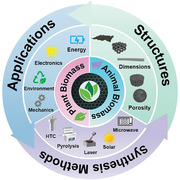

## 1 Introduction

Carbon materials have become an increasingly important research field due to their versatile structures, properties, and great potential in various applications, such as electrocatalysts,^[^ ^1^ ^]^ soft electronics,^[^ ^2^ ^]^ biomedical materials,^[^ ^3^ ^]^ and pollution treatment.^[^ ^4^ ^]^ However, the major precursors for carbon materials at present are fossil fuel derivatives such as methane, ethylene, asphalt, and polyacrylonitrile (PAN), which are unsustainable materials. In addition, due to the harsh and energy consuming synthesis process, the cost of carbon products produced using these sources are relatively high. Therefore, using cost‐effective, abundant, and sustainable carbon sources as substitutes for petroleum‐ and coal‐derived carbon precursors is highly desirable.

Biomass is a renewable hydrocarbon resource that can be obtained from a range of industries including agriculture, forestry and stock farming, and also from urban waste.^[^ ^5^ ^]^ Compared with fossil fuel‐based carbon precursors, biomass as the precursor for the preparation of carbon materials has the advantages of low cost, sustainable supply, extensive availability, and easy accessibility. It is noteworthy that biomass waste is the most valuable carbon source among all biomass sources considering its environmental benefits. For example, the world leather industry produces 8–9 MT of skin per annum and 1.4 MT of solid waste containing mainly protein is produced.^[^ ^8^ ^]^ Most of this solid biomass waste is discarded in the environment using either landfilling or thermal incineration. Disposal of this waste into the environment not only affects the ecosystem, causing air pollution (emitting odor) but also occupies land resources. However, using biomass waste as a carbon precursor can convert this otherwise valueless waste into high‐value functional carbon materials.^[^ ^6^, ^7^ ^]^

In addition to protecting the environment and reducing costs, using biomass as a carbon source has many other advantages. Due to its high carbon content and diverse molecular structures, biomass can be made into carbon materials with different structures, allowing these materials to be applied in versatile applications. For instance, carbon aerogels can be used as microwave absorption materials,^[^ ^9^ ^]^ while carbon dots can be applied for bioimaging.^[^ ^10^ ^]^ In addition, biomass molecules consist of various heteroatoms, such as S, N, and P. Thus the surface chemistry and properties of biomass‐derived carbon materials (BCMs) are quite different from the surface chemistry and properties of petroleum or coal‐derived carbons, thus offering different wettability, conductivity or chemical stability for particular applications.^[^ ^11^ ^]^

To realize the controllable preparation of BCMs, many fabrication approaches have been reported. Hydrothermal carbonization (HTC) and pyrolysis are the most widely used ways to prepare BCMs. Other methods, such as laser‐ and microwave‐assisted carbonization and solar‐induced graphitization, have also been adopted in recent years. Through these different preparation methods, as‐obtained BCMs often possess various morphologies and structures with diverse dimensions. In addition, using additives such as activation reagents and templates has a great effect on the porosity of BCMs, resulting in a highly porous structure.^[^ ^12^, ^13^ ^]^

Due to the complex chemical composition of biomass precursors, the thermal transformation mechanism of biomass is complicated. However, this process has a major influence on the quality and properties of the prepared BCMs. In general, several reactions occur during the carbonization process of biomass, including dehydration, decarboxylation, depolymerization, isomerization, and aromatization.^[^ ^14^, ^15^ ^]^ Numerous efforts have been devoted to probing the thermal transformation of biomass.^[^ ^16^ ^]^ Animal‐based and plant‐based biomasses normally show different pyrolysis behaviors. The former usually loosen the original biomass structure, while the latter can retain it.^[^ ^17^, ^18^, ^19^, ^20^ ^]^ Thus, it is critical to choose the right biomass source to obtain carbon materials with the desired structures and morphologies. Moreover, the rational design of synthesis is vital in order to obtain BCMs with targeted properties, including electric conductivity, mechanical resistance, and adsorption capacity. To build a powerful platform for data‐oriented experimental design of BCMs synthesis, machine learning methods have been developed in recent years to build.

**Figure 1:**
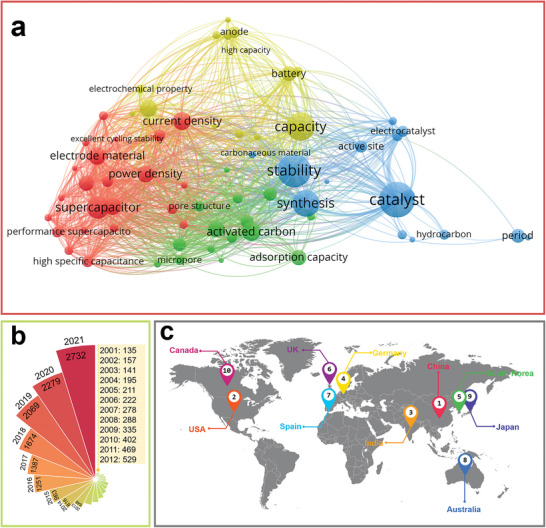
a) Key words network visualization of BCMs created by VOSviewer software using bibliographic data from Web of Science (keyword occurrence over 60 and relevant value of 60%). b) Annual number of published papers containing “biomass derived carbon” from 2011 to 2021. c) Top 10 research productive countries of BCMs according to Web of Science.

With diverse structures and surface chemistry, BCMs have shown great potential in versatile applications in energy,^[^ ^21^, ^22^ ^]^ electronics,^[^ ^23^, ^24^ ^]^ the environment,^[^ ^25^, ^26^ ^]^ and other fields, such as reinforcement materials.^[^ ^27^ ^]^ Based on the analysis of keywords of BCMs related research papers via VOSviewer (**Figure** **1**a), applications in the energy field including supercapacitors, metal ion batteries, and electrocatalysts are of great interest. The applications of BCMs are largely dependent on their structure and surface properties. For example, BCMs applied as electrocatalysts and electrode materials normally have porous structures because a well‐developed pore network is beneficial for exposure of active sites and diffusion of reactants and electrolytes.^[^ ^28^ ^]^ In addition, the surface chemical properties of BCMs are conducive to interfacial chemical reactions.^[^ ^29^ ^]^ BCMs with a high degree of graphitization are stiff and highly conductive and can be used as reinforcement fillers for polymer nanocomposites.^[^ ^30^ ^]^ Thus, the structure design is critical to improve the performance of BCMs.

**Scheme 1:**
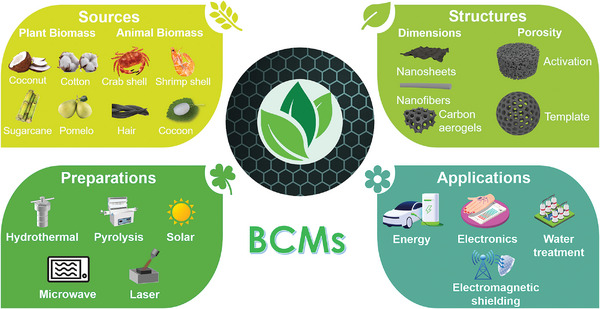
Various sources, structures, preparation methods, and versatile applications of BCMs.

As stated above, the conversion of biomass into high value‐added carbon products has significance considering its benefits with respect to waste management, biomass valorization, and low carbon emissions.^[^ ^31^, ^32^ ^]^ Indeed, research on BCMs has received increasing attention and is experiencing flourishing moments as shown by the increasing publication numbers from 2001 to 2021 in Figure 1b. Research into the development and applications of BCMs is conducted worldwide with countries such as China, USA, India, Germany, and South Korea leading the work in this field, as shown in Figure 1c in which the top 10 productive countries are listed. Considering the fast development and increasing research interest in BCMs, a timely and comprehensive review is essential. In this review, the common biomass precursors, including animal biomass and plant biomass, are introduced, and then typical structural features of BCMs, including the dimensions and porosity, are systematically summarized. In addition, various synthetic strategies are examined in detail. In the last section, recent progress in the applications of BCMs in the fields of energy, electronics, and environmental studies is reviewed. The main content of this review is summarized in **Scheme** **1**. This review paper provides a comprehensive overview of BCMs for researchers and aims to propel further development of BCMs.

## 2 Typical Biomass Precursors

**Figure 2:**
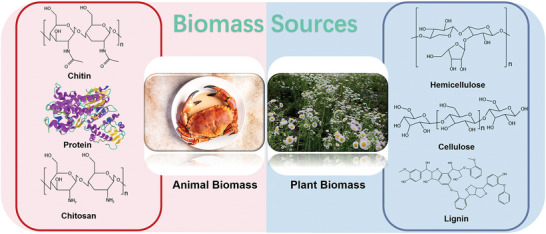
Summary of typical animal and plant biomass precursors for BCM preparation. Animal biomass precursors include chitin, protein, and chitosan. Plant biomass precursors include hemicellulose, cellulose, and lignin.

Biomass is the most abundant resource on earth.^[^ ^33^ ^]^ According to the definition given by the International Energy Agency, biomass is a biological material directly or indirectly produced by photosynthesis. Specifically, it includes organic products, wastes or residues from forestry, agriculture and households as well as livestock productions or wastes in animal husbandry. Biomass can be generated in a short cycle and is a renewable source. Apart from the rich resources, biomass is relatively cheap and easy to obtain since it is mostly waste materials. Broadly speaking, biomass precursors can be divided into two categories: Plant biomass and animal biomass. Plant biomass is usually synthesized through photosynthesis, and the main components are cellulose, hemicellulose, and lignin.^[^ ^34^ ^]^ Animal biomass refers to the waste or residue of animals and is composed of proteins, minerals, or polysaccharides such as chitosan and chitin.^[^ ^35^ ^]^ **Figure** **2** presents the two main groups of biomass sources, animal biomass, and plant biomass, that will be explored in the following sections of this review.

### 2.1 Plant Biomass

In this section, typical plant‐derived biomass precursors for BCMs are introduced, including macromolecular plant biomass (cellulose, hemicellulose, and lignin) as well as small molecule biomass (glucose and fructose).

#### 2.1.1 Cellulose

Cellulose is the most common macromolecule in plants.^[^ ^36^ ^]^ Up to 50% of the carbon element in the plant is contributed by cellulose. It constructs the skeleton of the cell wall in the form of bundles of fibers. Hydrogen bond and van der Waals interactions pack up the long chains of cellulose to generate microfibrils.^[^ ^37^ ^]^ Therefore, the molecular chains are crystalline and parallel, which endows cellulose with superior mechanical strength and strong mechanical properties. The cellulose molecule is composed of repeated units of glucose. Its molecular weight ranges from 50 000 to 2 500 000.

Cellulose has been widely used as a biomass precursor to prepare carbon materials. The resources of cellulose commonly used are either commercial cellulose or isolating cellulose from lignocellulose via the sulfite method and alkali method of extraction.^[^ ^38^, ^39^ ^]^ Pyrolysis is a convenient approach to fabricate cellulose‐derived carbon. Fan et al. studied the impact of the heating rate on the transformation of functional groups of cellulose into biochar during pyrolysis. The —OH group increased initially due to the cracking of glucose units and then decreased because of dehydration at higher temperature to form = C—H or C = O. The formation of C = C bonds occurred at high carbonization temperatures. In this period, —OH, C—C, and C = O bonds broke due to dehydration, dehydrogenation and/or cracking reactions. They also found that oxygen‐containing functional groups such as C—O—C remained stable during pyrolysis, which was the primary reason for the existence of oxygen in the final product.^[^ ^40^ ^]^ Gao et al. prepared porous flexible carbon paper with high conductivity from cellulose paper by a facile O_2_—NH_3_ reactive pyrolysis method. Moreover, the cellulose fiber in the paper was converted into ultrathin twisted carbonaceous sheets.^[^ ^41^ ^]^

**Figure 3:**
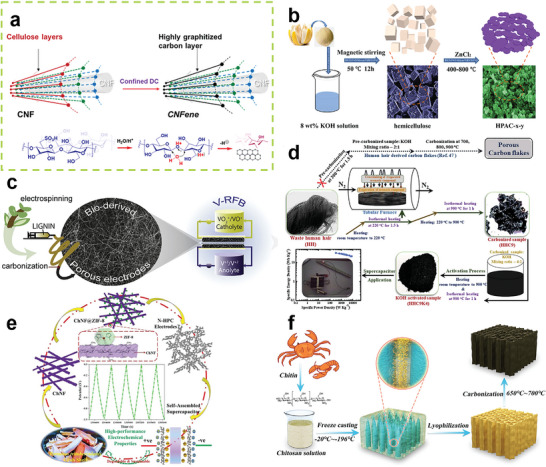
a) The structure transformation process of the nanosurface of CNF. Reproduced with permission.^[^ ^42^ ^]^ Copyright 2021, ACS. b) The preparation process of porous carbon derived from hemicellulose from pomelo peel. Reproduced with permission.^[^ ^48^ ^]^ Copyright 2019, Elsevier. c) The fabrication process of lignin‐based carbon fibers. Reproduced with permission.^[^ ^56^ ^]^ Copyright 2019, Elsevier. d) The preparation process of hierarchically porous heteroatom‐doped carbon from wasted human hair. Reproduced with permission.^[^ ^79^ ^]^ Copyright 2020, Elsevier. e) The fabrication process of porous carbon electrodes from chitin. Reproduced with permission.^[^ ^85^ ^]^ Copyright 2020, Elsevier. f) The preparation process of lightweight and 3D conductive carbon aerogels from chitosan. Reproduced with permission.^[^ ^91^ ^]^ Copyright 2021, Elsevier.

Different from traditional methods such as pyrolysis, which usually require high temperatures, Wang et al. proposed a new research perspective that is able to convert confined ranges of cellulose fibers into highly graphitized carbon at only 90 °C and atmospheric pressure. After the exaction of cellulose nanofiber (CNF) from biomass, a confined dehydration and carbonization reaction was continuously performed by using sulfuric acid under completely isolated oxygen conditions. The nanosurface of CNF was then transformed to highly graphitized carbon.^[^ ^42^ ^]^ The structure transformation process is illustrated in **Figure** **3**a.

#### 2.1.2 Hemicellulose

Hemicellulose is one of the most abundant polysaccharides in nature, accounting for 25–35% of lignocellulose biomass.^[^ ^43^ ^]^ Accompanied by pectin and cellulose, hemicellulose constructs the cell wall of plants.^[^ ^44^ ^]^ Unlike cellulose, hemicellulose has amorphous structures because of its diverse polysaccharide structural units. Pentoses (arabinose and xylose), hexoses (galactose, glucose and mannose), and hexuronic acids (glucuronic acid) are common building blocks of hemicellulose.^[^ ^45^ ^]^ The nomenclature of hemicellulose is determined by the main sugar units in the molecular backbone.^[^ ^44^ ^]^ Because of its solubility in alkaline solutions, hemicellulose is usually extracted from biomass by alkali treatments.^[^ ^46^ ^]^ Hemicellulose has a smaller degree of polymerization than that of cellulose and is a “soft tissue,” which endows the carbon products derived from hemicellulose with special properties. Wang et al. chose hemp stem hemicellulose as the precursor and converted it into carbon materials through low‐temperature HTC and KOH activation. The as‐prepared carbon materials have a well‐shaped spherical morphology and a large specific area of 3062 m^2^ g^−1^.^[^ ^47^ ^]^ Lin et al. extracted hemicellulose from pomelo peel, and the precursor was carbonized with zinc chloride activation. The preparation process is shown in Figure 3b. After activation, the carbon material has a porous morphology with a large specific area of 1361 m^2^ g^−1^.^[^ ^48^ ^]^

#### 2.1.3 Lignin

Lignin is a natural aromatic polymer on earth with an amorphous 3D macromolecule.^[^ ^48^ ^]^ Methoxylated phenylpropanoid units are connected by ether bonds and carbon–carbon bonds.^[^ ^49^ ^]^ Lignin can enhance the rigidity and nonperishable properties of the plant. Lignin can be extracted from plants via the solvent method,^[^ ^50^ ^]^ the acid method,^[^ ^51^ ^]^ and the alkaline method.^[^ ^52^ ^]^ At present, most lignin resources are treated as pollutants and are usually discarded.^[^ ^53^ ^]^ Therefore, using lignin as the biomass precursor to prepare carbon materials is considered a high value‐added utilization.^[^ ^54^ ^]^ Lignin‐derived fine fibers were prepared by You et al. through electrospinning. Porous carbon fibers were further fabricated by thermal stabilization, carbonization, and steam activation, and the specific surface area (SSA) was up to 1880 m^2^ g^−1^. The carbon fiber showed potential applications in electrode fabrication. Due to the large SSA and small pore diameter, the carbon fiber electrodes prepared under optimized processing parameters have a specific capacitance of 92.6 F g^−1^ at a scan rate of 1 A g^−1^ and an impedance of 1.6 Ω.^[^ ^55^ ^]^ Ribadeneyra et al. also used the electrospinning method to prepare lignin‐based carbon fibers as sustainable electrodes for all‐vanadium flow batteries. Figure 3c is a schematic of the fabrication process. The carbon fiber has a diameter of 0.9–1 µm.^[^ ^56^ ^]^

Apart from the aforementioned macromolecular biomass, functional BCMs have been prepared from small molecules including glucose^[^ ^57^ ^]^ and fructose.^[^ ^58^ ^]^ Compared with macromolecular biomass, these simple biomass precursors can be synthesized with regulated morphology via HTC under mild conditions. When polymers, such as polysaccharides, undergo the hydrolysis process, they can form monosaccharides. The corresponding HTC requires severe conditions, for example, high reaction temperatures and often leads to the formation of uneven carbon products.^[^ ^59^ ^]^ Wang and co‐workers have reported the controllable synthesis of hydrochar derived from small molecular biomass with specific morphologies, such as flowers,^[^ ^60^ ^]^ hollow nanoflasks,^[^ ^61^ ^]^ hollow bowls,^[^ ^62^ ^]^ bivalves,^[^ ^63^ ^]^ and mesoporous nanowires.^[^ ^64^ ^]^ By adopting different means of regulation of the hydrothermal process, they can control the synthesis of BCMs from the microscale, the nanoscale, and even the molecular level.

#### 2.1.4 Glucose

Glucose is the most common monosaccharides in nature and is the basic structural unit (BSU) of several polysaccharides such as starch and cellulose. Zhang et al. reported a facile method for the large‐scale production of high‐quality graphene using glucose as the renewable feedstock and FeCl_3_ as the template and catalyst.^[^ ^65^ ^]^ This simple synthesis route composes the dissolution of glucose and FeCl_3_ in water, water vaporization in air and calcination at 700 °C. The obtained graphene sheets consist of few layers and their electrical conductivity (768 S m^−1^) is close to that of the graphene prepared by the chemical vapor deposition (CVD) method (1000 S m^−1^).^[^ ^66^ ^]^ With tunable structures, hydrochars derived from small molecular biomass are used as heat‐ and water‐tolerant supports for metal nanoparticles.^[^ ^57^ ^]^ For example, Rey‐Raap et al. used glucose‐derived carbon as the support for ruthenium metal for the catalytic conversion of cellulose to sorbitol.^[^ ^67^ ^]^ The chemical and textural properties of glucose‐derived carbon support were tailored by activation and addition of carbon nanotubes (CNTs). The results indicate that carbon supports with high microporosity and low acidity are beneficial to the dispersion of metal particles.

#### 2.1.5 Fructose

Fructose is the isomer of glucose and exists in honey and fruits. Hydrochars derived from fructose have been widely used as the carbon support for metal nanoparticles. Compared with other hexoses such as d‐glucose, the carbonization temperature of fructose is lower (130 °C for fructose and 150 °C for d‐glucose).^[^ ^58^ ^]^ Heckel et al. synthesized fructose derived carbon shells around silver nanoparticles with different thickness via HTC.^[^ ^68^ ^]^ The carbon shell not only can protect and stabilize Ag nanoparticles in high salt concentration but also can minimize the damping effect on the plasmon resonance. Besides, the size, the rate of growth and the degree of carbonization can be tuned by adjusting the fructose concentration, reaction time, and reaction temperature. Carbon support derived from fructose can be functionalized with various functional groups for better performance. Mahyari et al. found that fructose‐derived carbon supports functionalized by thiol groups can enhance the stability and eliminate the leaching of the loaded Au nanoparticles.^[^ ^58^ ^]^ In the recent research of Kurniawan et al., fructose was used to fabricate graphene quantum dots (GQDs) as environmental nanoprobes.^[^ ^69^ ^]^ The conversion process of fructose occurred under the ambient condition with the assistance of microplasma. The as‐prepared GQDs have an average size of 4.5 ± 1.7 nm and a broad size distribution with a deviation of ≈38%. To achieve the controllable synthesis of biomass with small molecules, the reaction mechanism of hydrothermal method and the influence of processing parameters are significant. These contents will be discussed in the following chapter.

### 2.2 Animal Biomass

Animal biomass contains more complex components than plant biomass. In this section, several animal biomass precursors which are easily obtained and commonly used for BCMs preparation are summarized.

Protein is one of the essential nutrients of organisms. It is an indispensable component of cells and tissues. Amino acids are the basic units for proteins, and they are linked together to form peptides via dehydration‐condensation reactions. All the proteins contained nitrogen elements, and the average content of nitrogen was 16%. To satisfy people's intake of proteins, more than 300 million tons of animal meats are produced per year worldwide.^[^ ^70^ ^]^ This process also generates large quantities of protein byproducts. Unlike carbohydrates, which can be converted into short chain alcohols and biodiesels, proteins are difficult to use for the production of biofuels due to the difficulty of deamination of protein hydrolysates.^[^ ^71^ ^]^ Most of the byproducts are wasted directly. However, due to their N‐rich properties, proteins are promising biomass precursors for the in situ synthesis of N‐doped carbon materials, which have been proven to have enhanced electrochemical properties compared to pure carbon materials.^[^ ^72^ ^]^ There have been many attempts to synthesize protein‐derived carbon materials using various protein‐rich biomasses, such as milk,^[^ ^73^ ^]^ silk fibroin,^[^ ^74^ ^]^ egg,^[^ ^75^, ^76^ ^]^ soybean,^[^ ^77^ ^]^ and collagen.^[^ ^78^ ^]^ Keratin, existing in human and animal hair and nails, is one of the common proteins in daily life. Sinha et al. used wasted human hair to synthesize hierarchically porous heteroatom‐doped activated carbon nanosheets for high‐performance supercapacitor electrodes. As shown in Figure 3d, a modified carbonization process with a hold of 1.5 h at 220 °C is proposed to prevent the evaporation of volatile molecules and to promote the crosslinking of heteroatoms as well as to rearrange the heteroatoms. A similar strategy was adopted in the activation process to decrease heteroatom loss.^[^ ^79^ ^]^

Chitin is a kind of linear polysaccharide polymer and is the second most abundant biopolymer on earth after cellulose. The molecular structure is illustrated in Figure 2. Up to 3000 acetylglucosamines are connected together via the *β*‐1,4 glycoside chain, and thus, the molecular weight can be as high as one million. Due to the strong intermolecular hydrogen bonds, chitin is insoluble in water and alkali solutions. Chitin is the primary component of cuticles or crustacean shells and cell walls of fungi.^[^ ^80^ ^]^ The chitin content in shrimp shells is up to 36.43%.^[^ ^81^ ^]^ Chitin has been widely applied in biomedical fields as a wound dressing^[^ ^82^ ^]^ and suture^[^ ^83^ ^]^ because of its good biocompatibility and nontoxic property. With millions of tons of seafood waste discarded annually, it is meaningful to seek new ways to utilize this chitin source for sustainable development.^[^ ^84^ ^]^ Shang et al. extracted chitin from crab shells to prepare chitin nanofibers as the framework for the in situ synthesis of ZIF‐8 to avoid fragmentation, as shown in Figure 3e. The nanocomposites were further carbonized and applied as porous carbon electrodes for supercapacitors.^[^ ^85^ ^]^ Chitin‐derived carbon materials have also been used in lithium‐ion batteries. Liu et al. exfoliated chitin powder to obtain chitin nanosheets. Then, a composite structure was constructed with nanosheets, polyvinyl alcohol and SiO*_x_*. After carbonization, the nanocomposites can be used as the anode for lithium‐ion batteries.^[^ ^86^ ^]^

Chitosan can be extracted from chitin after deacetylation.^[^ ^87^ ^]^ Chitosan has free amino groups and is the only natural alkaline polysaccharide. Chitosan can be made into various structures, including 3D gels and sponges, 2D films, and fibers.^[^ ^88^ ^]^ Chitosan is soluble in dilute acid solutions, including acetic acid, to form homogeneous hydrogel precursors. In addition, chitosan has a strong ability to absorb heavy metal ions.^[^ ^89^ ^]^ Freeze‐drying or casting chitosan solution is a common method to prepare porous chitosan‐based carbon. Duan et al. prepared chitosan‐derived carbon matrix‐encapsulated CuP_2_ nanoparticles for the anode of sodium‐ion batteries. The chitosan solution prevents the particle aggregation of CuP_2_.^[^ ^90^ ^]^ Tian et al. freeze‐casted a chitosan solution and obtained a chitosan aerogel. After carbonization, a lightweight and 3D conductive carbon aerogel can be fabricated, as shown in Figure 3f.^[^ ^91^ ^]^

## 3 Structures of Biomass‐Derived Carbon Materials

**Figure 4:**
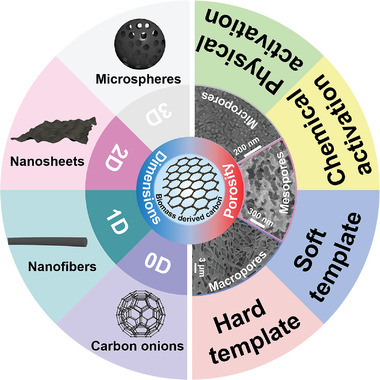
Schematic diagram of the diverse structures of BCMs. Left: The typical structure of BCMs with various dimensions. Right: Different pore structures of BCMs and synthesis methods of porous structures.

Due to the various biomass precursors and different synthesis methods, the obtained carbon materials have diverse structures. **Figure** **4** summarizes the different structures of BCMs. In this section, the typical dimensional structures and porous structures of BCMs are discussed.

### 3.1 Dimensions of Biomass‐Derived Carbon Materials

The performance of carbon materials is largely dependent on their structures. Different preparation methods can induce various internal structures.^[^ ^92^ ^]^ Due to the variety of molecules, BCMs can be designed into structures with multiple dimensions, including 0D, 1D, 2D, and 3D structures.^[^ ^93^, ^94^ ^]^ With the multifarious structures, BCMs can be used in a variety of applications. For instance, the photoluminescence nature of 0D BCMs enables their applications in bio‐imaging and nano‐medicine.^[^ ^95^, ^96^ ^]^ BCMs with the 1D structure are advantageous to energy storage devices such as supercapacitors because of their continuous electron transport path, good mechanical properties and high ion‐accessible surface area for charge aggregation.^[^ ^97^, ^98^ ^]^ 2D sheet‐like BCMs are considered as the ideal framework for electrodes in lithium‐ion batteries because of their short solid‐state diffusion distance for Li ions during charge and discharge processes in the confined spaces between stacks.^[^ ^99^ ^]^ 3D carbon materials usually exhibit superb performance in energy conversion and storage,^[^ ^100^ ^]^ gas adsorption,^[^ ^101^ ^]^ water treatment,^[^ ^102^ ^]^ and electromagnetic shielding,^[^ ^13^ ^]^ due to their inherent high SSA and well‐interconnected porous 3D structure.^[^ ^103^ ^]^ Thus, it is significant to design the dimensional structure of carbon materials as per the corresponding applications.

#### 3.1.1 0D Structure

0D carbon materials include carbon nanoparticles (CNPs),^[^ ^104^ ^]^ carbon nanodots (CNDs),^[^ ^105^ ^]^ carbon onions,^[^ ^106^ ^]^ and carbon nanocages (CNCs).^[^ ^107^ ^]^ Due to the small size, large SSA, and quantum size effects of 0D carbon materials, they usually exhibit special physical or chemical properties, such as good mobility and excellent conductivity, indicating promising applications in the energy and electronic fields.^[^ ^108^, ^109^ ^]^ However, 0D carbon materials require a complex preparation process, and it is difficult to obtain 0D carbon materials directly from biomass in nature.^[^ ^110^, ^111^ ^]^

**Figure 5:**
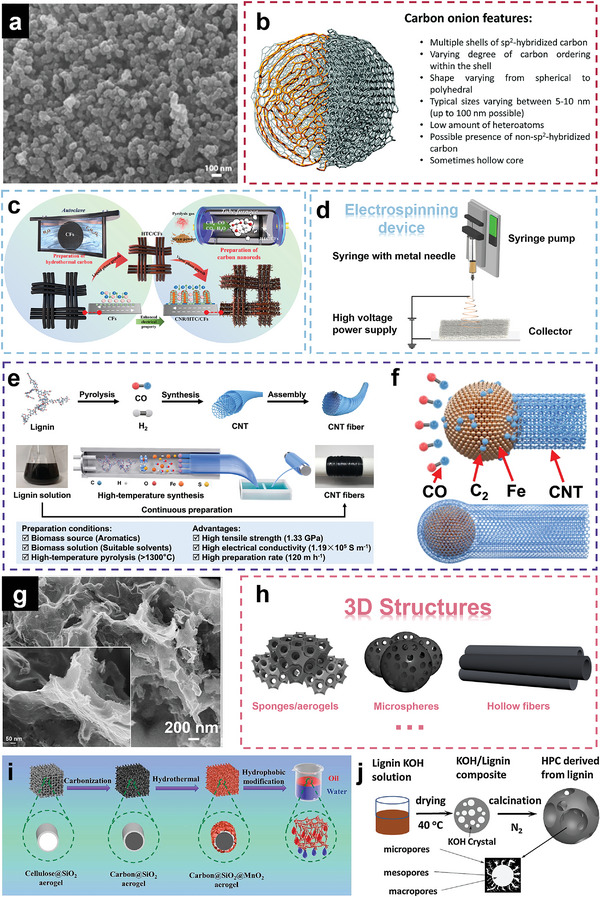
a) Morphology of CNPs derived from coconut oil. Reproduced with permission.^[^ ^112^ ^]^ Copyright 2016, Elsevier. b) Typical features of carbon onions. Reproduced with permission.^[^ ^106^ ^]^ Copyright 2016, RSC. c) The preparation process of straw‐derived carbon nanorod‐supported hydrothermal carbon and carbon fiber. Reproduced with permission.^[^ ^154^ ^]^ Copyright 2021, Elsevier. d) The schematic figure of a basic setup of the electrospinning device. e) Synthesis mechanism from lignin to CNT fibers and its respective processing schematic. Reproduced with permission.^[^ ^166^ ^]^ Copyright 2022, The authors, Springer Nature. f) Schematic of the “tap” type growth model of lignin derived CNTs. Reproduced with permission.^[^ ^166^ ^]^ Copyright 2022, The authors, Springer Nature. g) Morphology of carbon nanosheets derived from pine nuts. Reproduced with permission.^[^ ^183^ ^]^ Copyright 2019, ACS. h) Typical 3D structures of carbon materials. i) Fabrication process of cellulose‐derived carbon aerogels. Reproduced with permission.^[^ ^197^ ^]^ Copyright 2015, Elsevier. j) Flowchart of the synthesis process of 3D hierarchical porous carbon material derived from lignin. Reproduced with permission.^[^ ^198^ ^]^ Copyright 2015, Elsevier.

CNPs have been applied in energy storage materials such as lithium‐sulfur batteries (LSBs). CNPs are able to act as sulfur containers and enhance the conductivity of sulfur during cycling.^[^ ^104^ ^]^ Gaddam et al. prepared CNPs from coconut oil by a flame decomposition method. They used piranha solution to carboxylate the surface of CNPs. The coconut oil‐derived CNPs exhibited a quasi‐spherical morphology with particle sizes ranging from 40 to 50 nm,^[^ ^112^ ^]^ as presented in **Figure** **5**a. It was found that the surface chemistry of CNPs has a notable effect on the electrochemical performance.^[^ ^113^ ^]^ However, the exact mechanisms of the surface groups still need to be further investigated.

CNDs are spherical carbon materials with particle sizes less than 10 nm. They were first discovered by Xu et al.^[^ ^114^ ^]^ and then named by Sun et al. in 2006.^[^ ^115^ ^]^ Similar to other quantum‐sized materials, CNDs also have florescent properties and good photostability. Due to their small size and various functional groups on the surface, CNDs exhibit unique electronic properties and possess low toxicity, good chemical inertness and high electrochemical conductivity.^[^ ^116^, ^117^ ^]^ Consequently, CNDs have been applied in various fields, such as drug delivery,^[^ ^118^ ^]^ bioimaging,^[^ ^119^ ^]^ chemical sensors,^[^ ^120^ ^]^ and energy devices.^[^ ^121^ ^]^ Two approaches are commonly adopted for the preparation of CNDs: The top‐down method and the bottom‐up method. The former is cutting CNDs from as‐prepared carbon precursors by arc discharge,^[^ ^122^ ^]^ laser ablation.^[^ ^123^ ^]^ CNDs obtained through this method have a graphitic structure, but the synthesis process requires harsh conditions and complicated procedures. The bottom‐up method is to carbonize polymers or small molecules into CNDs. This fabrication process is cost‐effective and does not require harsh conditions, and the CNDs are usually amorphous.^[^ ^124^ ^]^ Biomass‐derived CNDs are synthesized mostly by the bottom‐up method. Successful preparation of CNDs from silk fibroin,^[^ ^125^ ^]^ bamboo leaves,^[^ ^126^ ^]^ pomelo peel,^[^ ^127^ ^]^ and gelatin^[^ ^128^ ^]^ has been reported, demonstrating the feasibility of the transformation of animal and plant biomass into CNDs.

Carbon onions were first discovered by Lijima in 1980^[^ ^129^ ^]^ and were described by Ugarte in 1992.^[^ ^130^ ^]^ The name was given by the multilayer structure of the material. Several spherical or polyhedral carbon shells are enclosed in the carbon onion layer by layer, similar to a Russian doll.^[^ ^131^ ^]^ The size of carbon onions ranges from 5 to 50 nm depending on various synthesis approaches.^[^ ^132^, ^133^ ^]^ Typical features of carbon onions are summarized by Zeiger et al., as shown in Figure 5b.^[^ ^106^ ^]^ Han et al. fabricated nano‐onion‐like carbon derived from Lentinus edodes as photocatalysts.^[^ ^134^ ^]^ The size of the carbon onion is ≈20–30 nm with 2–5 layers.

CNCs possess a hollow structure with diverse shapes such as quasi‐sphere^[^ ^135^ ^]^ and cubes,^[^ ^136^ ^]^ and are formed by the crimp of carbon layers.^[^ ^137^, ^138^ ^]^ As an emerging member in the carbon nanomaterial family, CNCs have unique structural features such as large interior cavities, smooth mass and charge transfer scaffold, tunable electronic structure as well as large SSA as summarized by Wu et al.^[^ ^139^ ^]^ With these structural features, CNCs exhibit great potential in the applications such as energy storage materials (supercapacitors,^[^ ^140^ ^]^ LSBs^[^ ^141^ ^]^) and energy conversion materials (electrocatalysts for oxygen reduction reaction (ORR),^[^ ^142^ ^]^ oxygen evolution reactions (OERs),^[^ ^143^ ^]^ and hydrogen evolution reaction (HER)^[^ ^144^ ^]^). Zhao et al. prepared CNCs from spring onion peel via an one‐step strategy for both supercapacitors and sodium‐ion batteries.^[^ ^145^ ^]^ The as‐prepared nanocages have uniform morphology with a size in the range of 50–80 nm and a 4 nm thick porous wall. The SSA of the biomass‐derived CNCs is up to 1776 m^2^ g^−1^, and the pore volume is calculated to be 2.2 cm^3^ g^−1^.

#### 3.1.2 1D Structure

Carbon materials with 1D structures have the advantages of a high SSA, excellent conductivity and good flexibility.^[^ ^146^ ^]^ Because of these properties, 1D carbon materials are widely applied in energy devices^[^ ^147^ ^]^ and electronics.^[^ ^148^ ^]^ In addition, 1D carbon materials can be used as supports for metal nanomaterials to construct high‐performance nanocomposites.^[^ ^149^ ^]^ The 1D design of carbon materials has various morphologies, including nanorods, nanotubes, and nanofibers.

##### Carbon Nanorods

Carbon nanorods have attracted considerable interest for their potential as anodic materials in secondary batteries.^[^ ^150^ ^]^ Some biomolecules can self‐assemble into nanorods, such as alizarin.^[^ ^151^ ^]^ The self‐assembly process is achieved due to the different solubilities in acetone and water of the aromatic alizarin molecule. Specifically, alizarin was dissolved in acetone and then dropped into the water where alizarin was not soluble, which triggered the recrystallization and self‐assembly of the alizarin molecule. In this period, the *π*–*π* interaction and hydrogen bonding played a critical role.^[^ ^152^ ^]^ Most of the assembly direction was vertical to the molecular plane, and oriented growth formed the nanorod structure.^[^ ^153^ ^]^ Fang et al. fabricated carbon nanorod‐supported hydrothermal carbon and carbon fiber derived from the pyrolysis gas of straw.^[^ ^154^ ^]^ The construction of carbon nanorods relied on the vapor phase growth strategy, as shown in Figure 5c.

##### Carbon Nanofibers

CNFs have a high SSA and superb charge conduction ability, which can provide more active sites in electrochemical reactions.^[^ ^155^ ^]^ Thus, CNFs can be highly effective energy storage materials. Although there are many fibrous biomasses in nature, the smallest sizes are on the micrometer scale, for example, silk fibers, cotton fibers, and wool fibers. The priority of the preparation of biomass‐derived CNFs is to fabricate nanofibers from the biomass precursors. Electrospinning technology is considered an effective approach to prepare fibrous materials at the nanoscale.^[^ ^156^ ^]^ It has several advantages, such as low spinning cost and various choices of spinning substances. In addition, the spinning process and the morphology of the fibers are controllable by adjusting the spinning parameters, such as the voltages, injection speed, and collection distance. The spinning devices can also be redesigned to obtain special structures, such as skin‐core structures and hollow fibers.^[^ ^157^ ^]^ The basic setup of the electrospinning device normally includes four components: A high voltage power supply, a syringe with a metal needle, a syringe pump and a collector (either a metal plate or a rolling cylinder), as illustrated in Figure 5d. Our group has prepared porous CNFs from silk fibroin by electrospinning and carbonization.^[^ ^158^ ^]^ The diameter of the silk fiber is 25 µm, while that of silk fibroin electrospun nanofibers is 447.25 ± 66.45 nm, indicating the slump of the fiber size due to electrospinning.

##### Carbon Nanotubes

CNTs are one of the most studied 1D carbon nanomaterials of the tubular structure whose diameter is in nanoscale while the length can be millimeters.^[^ ^159^ ^]^ CNTs are typically prepared through several methods, such as arc‐discharge method,^[^ ^160^ ^]^ CVD,^[^ ^161^ ^]^ and pyrolysis,^[^ ^162^ ^]^ using petroleum and coal products including benzene, methane, and ethylene with the catalysis of Fe, Ni, and Co, etc.^[^ ^5^ ^]^ Efforts have been devoted to the preparation of CNTs using biomass as a renewable feedstock for over a decade. Zhou et al summarized the recently reported preparation methods, including plant biomass (grass, chlorella, corn), bio‐extracts (D‐glucosamine hydrochloride, chitosan, cotton fiber) and bio‐wastes (sawdust, potato peels, paper sludge).^[^ ^163^ ^]^

Moreover, the existence of oxygen within biomass facilitates the oxidization of amorphous carbon, which improves the nucleation process of CNTs.^[^ ^164^ ^]^ Besides, the H_2_O molecules produced by the decomposition of biomass are beneficial to prevent the oxidization or the damage of CNTs by eliminating the amorphous carbon.^[^ ^165^ ^]^ Liu et al. reported the continuous fabrication of CNT fibers from waste lignin by solvent dispersion, high‐temperature pyrolysis, catalytic reaction, and assembly.^[^ ^166^ ^]^ The production process is illustrated in Figure 5e. The production rate of lignin‐derived CNT fiber is up to 120 m h^−1^ and CNT fiber possesses a high tensile strength of 1.33 GPa as well as an electrical conductivity of 1.19 × 10^5^ S m^−1^. For the formation mechanism of lignin derived CNTs, CO released by the decomposition of lignin during pyrolysis first adsorbs on the surface of iron catalyst particles, and C—C dimers are formed. Afterward, these dimers leave the catalyst surface and link with other dimers to form short chains. At last, sp2 bonded graphene sheets will be formed by the connection of short chains. The growth model of lignin‐derived CNTs is called the “tap” type which means all layers of the CNTs are deposited on the surface of the catalysts at the same time as shown in Figure 5f.

#### 3.1.3 2D Structure

BCMs with the 2D structure are normally lamellar and with abundant sp^2^ hybridizations.^[^ ^29^, ^167^ ^]^ 2D BCMs have several advantages for application in energy storage and conversion applications. First, due to their strong in‐plane covalent bonds, 2D BCMs possess high in‐plane conductivity.^[^ ^168^ ^]^ Second, 2D BCMs have a large flat surface which not only provides a large SSA but also is beneficial for the exposure of active sites for electrochemical reactions.^[^ ^169^ ^]^ Third, 2D BCMs have rich active surface edge and in‐plane defect active sites to facilitate electrochemical processes such as charge storage.^[^ ^170^ ^]^ Carbon nanosheets are typical morphology of 2D BCMs and “nano” refers to the magnitude of the sheet thickness. Ideally, nanosheets consist of single monolayer, but they often comprise a small number of stacked layers (normally < 10), manifested as incompletely exfoliated flakes.^[^ ^171^ ^]^ According to the internal carbon structure, such carbon nanosheets can be categorized into graphene and graphene‐like carbon.

Graphene is the most well‐known 2D carbon material and is composed of sp^2^‐hybridized carbon atoms arranged in a honeycomb lattice.^[^ ^172^ ^]^ Besides carbon, biomass often contains large amounts of other elements, such as hydrogen and oxygen. Therefore, to prepare graphene from biomass, dehydration and crystallization of biomass at a high temperature are necessary.^[^ ^163^ ^]^ These processes can align and assemble biomolecules into layered structures. Graphene is formed by these structures during carbonization. Yuan et al. reported a scalable synthesis method of few‐layer graphene from biomass via a solvent‐free approach called the “shearing/graphitization process.”^[^ ^173^ ^]^ In brief, biomass and FeCl_3_·6H_2_O were first shear mixed in a kitchen blender and then the mixture was pre‐carbonized and graphitized at 450 °C and 1000 °C, respectively. During shearing, biomass was exfoliated to form dispersed flake microstructure and impregnated with catalytic iron species. Subsequently, during the catalyzed carbonthermal process, biomass was graphitized into micron‐scale graphene flakes. This preparation method can be applied to various biomass precursors including saw dust, coconut husk, peanut shell, corncob, and tea leaves. Chen et al. prepared few‐layer graphene from waste wheat straw via a combined hydrothermal and graphitization process.^[^ ^174^ ^]^ The obtained graphene has 2–10 atomic layers and a mesoporous structure. Chen et al. used the CVD method to prepare graphene from cellulose acetate.^[^ ^175^ ^]^ It was found that cellulose acetate first evolved to reduced graphene oxide and then to mono‐layer graphene. To transform graphene flakes into few‐ or single‐layer graphene, exfoliation is necessary. Sonication is a facile way for exfoliation which uses sound energy to break interlayer interactions of stacked graphene. Purkait et al. obtained few‐layer graphene from peanut shells using a probe‐sonication sulfuric acid solution.^[^ ^176^ ^]^ Using template is another effective way to obtain few‐layer or monolayer graphene. Li et al. chose graphitic carbon nitride (g‐C_3_N_4_) as the template to obtain graphene from glucose.^[^ ^177^ ^]^ During graphitization, g‐C_3_N_4_ decomposes into NH_3_, C_2_N_2_^+^, C_3_N_2_^+^, and C_3_N_3_^+^ species and thus it can dope N into the resulting graphene. This method can be used to produce monolayer or two‐layer graphene.

The carbonization process involves the transformation of sp^3^ hybridized carbon to aromatic sp^2^ hybridized carbon. However, the carbonization of biomass often derives amorphous carbon, instead of graphene.^[^ ^163^ ^]^ Although some prepared biomass carbon materials may exhibit layered structure, they usually contain disordered carbon structure, defects (vacancies, sp^3^ hybridized carbon, oxygenated functions, etc.) and have low graphitization degree. These carbon materials are defined as graphene‐like materials.^[^ ^178^ ^]^ They show most properties of graphene but they are not perfect graphene. Biomass such as soybean milk,^[^ ^179^ ^]^ melaleuca bark,^[^ ^180^ ^]^ willow catkin,^[^ ^181^ ^]^ and pomelo peels^[^ ^182^ ^]^ can be converted into graphene‐like carbon nanosheets via direct carbonization. Guan et al. fabricated porous carbon nanosheets from pine nuts through carbonization together with KOH and melamine activation.^[^ ^183^ ^]^ The morphology of pine nut‐derived carbon nanosheets is shown in Figure 5g. This 2D nanomaterial has a large SSA up to 2093 m^2^ g^−1^, with 2036 m^2^ g^−1^ contributed by micropores. Liu et al. reported a one‐step method for the synthesis of graphene‐like porous carbon nanosheets from naturally layered peanut seed coats via simultaneous thermal‐exfoliation and pyrolysis.^[^ ^184^ ^]^ The biomass precursor was pre‐treated by intercalating triethanolamine into the interlayer. The obtained carbon nanosheets have a thickness of ≈4 nm and a conductivity of 8.7 S cm^−1^. **Table** **1** summarizes several graphene and graphene‐like 2D BCMs.

**Table 1:** A summary of graphene and graphene‐like 2D BCMs

Biomass	Method	Product	Thickness	Lateral size	Yield	Conductivity	References
Saw dust	Solvent‐free shearing/graphitization process	Graphene flakes	2–10 layers	0.5–15 µm	17.83 ± 2.63 wt%	1.39 × 10^4^ S m^−1^	[173]
Wheat straw	KOH chemical activation and pyrolysis	Few‐layer graphene	2–10 layers	3 µm	/	/	[174]
Cellulose acetate	CVD	Graphene	Monolayer	106–1593 nm	/	/	[175]
Peanut shell	Pyrolysis and probe sonication	Few‐layer graphene	6–8 layers	/	/	/	[176]
Glucose	Template confinement and pyrolysis	Monolayer graphene	Monolayer	/	/	785 S m^−1^	[177]
Corn cob	Combustion into biochar and then electrochemical exfoliation	Graphene	2 layers	7.70 µm	/	114.5 S m^−1^	[185]
Pomelo	Hydrothermal and carbonization	Graphene‐like porous carbon nanosheets	11–410 nm	/	/	/	[182]
Peanut seed coats	Triethanolamine intercalation and pyrolysis	Graphene‐like porous carbon nanosheets	4 nm	/	/	810 S m^−1^	[184]
Walnut shell	Carbonization and KOH chemical activation	Graphene‐like porous carbon nanosheets	5–6 layers of graphene nanosheets	/	21 wt%	/	[186]
Glucose, fructose and 5‐hydroxymethylfurfural	Pyrolysis	Graphene‐like carbon nanosheets	/	/	3.6–6.7 wt%	/	[187]
Almond shells	Pyrolysis and KOH activation	Graphene‐like carbon nanosheets	2 layers of carbon sheets	/	/	/	[188]

#### 3.1.4 3D Structure

The 3D design of carbon materials has attracted great research interest due to the advantages of 3D structures: excellent interconnectivit, large SSA, tailorable porous structures, both mechanical and chemical stability, and low density.^[^ ^189^, ^190^, ^191^, ^192^ ^]^ These features are beneficial to enhance the material performance in environmental applications, such as gas adsorption, wave absorption, and water treatment. Electrical properties also play a key role in applications such as electrocatalysts, soft electronics, and electrochemical energy storage devices (EESDs). While 3D carbon structures assembled from graphene, CNTs, or carbon fibers have high electrical conductivity inherited from graphene and CNTs and can act as conductive scaffold.^[^ ^193^, ^194^ ^]^ Typical 3D carbon materials include carbon sponges or aerogels, carbon microspheres, and carbon hollow fibers, as presented in Figure 5h. It is noteworthy that the construction of 3D architecture from biomass materials usually requires extra procedures, among which freeze‐drying is the most common approach.^[^ ^195^ ^]^

Li et al. fabricated porous carbon aerogels from silk cocoons via freeze‐drying and pyrolysis.^[^ ^196^ ^]^ Silk fibroin was extracted from silk cocoons by degumming, dissolution, and dialysis, and then was dissolved with FeCl_3_. The solution was further freeze‐dried and carbonized to obtain N, S, and Fe ternary‐doped carbon aerogels. The porous structure of the aerogel contributed a large specific area of up to 714.4 m^2^ g^−1^. Yuan et al. prepared sisal cellulose‐derived carbon aerogel composites for oil–water separation.^[^ ^197^ ^]^ They first used the sol–gel method to obtain cellulose@SiO_2,_ and the precursor was then freeze‐dried to obtain an aerogel. After carbonization and hydrothermal processes, carbon@SiO_2_@MnO_2_ aerogel was synthesized. Figure 5i shows the preparation process of the material. The composites are flexible and have strong mechanical strength due to the cellulose framework of the carbon aerogel. Zhang et al. used lignin as the biomass precursor to prepare 3D hierarchical porous carbon.^[^ ^198^ ^]^ KOH was used as the active reagent to construct the 3D network. After KOH activation, the carbon material possesses a 3D hierarchical porous structure with macropores as the carbon skeleton, micropores, and mesopores decorated on the carbon wall, as shown in Figure 5j.

### 3.2 Porosities of Biomass‐Derived Carbon Materials

#### 3.2.1 Hierarchical Porous Structure

**Figure 6:**
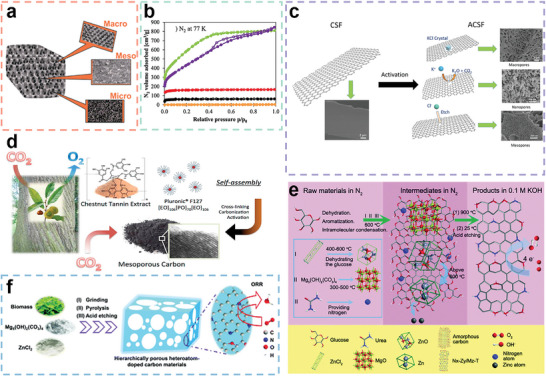
a) Illustration of micropores, mesopores and macropores. Reproduced with permission.^[^ ^200^ ^]^ Copyright 2014, RSC. b) Adsorption/desorption isotherms of nitrogen at 77 K. Reproduced under terms of the CC‐BY license.^[^ ^201^ ^]^ Copyright 2021, The authors, published by Elsevier. c) Hierarchical porous structure of silk fibroin‐derived carbon materials by KCl activation. Reproduced with permission.^[^ ^74^ ^]^ Copyright 2021, Elsevier. d) Preparation process of chestnut tannin‐derived mesoporous carbons by the soft template method. Reproduced with permission.^[^ ^202^ ^]^ Copyright 2015, Elsevier. e) Schematic figure of the dual‐templating method proposed by Li et al. Reproduced with permission.^[^ ^203^ ^]^ Copyright 2019, RSC. f) Illustration of the templating mechanism of Mg_5_(OH)_2_(CO_3_)_4_/ZnCl_2_. Reproduced with permission.^[^ ^203^ ^]^ Copyright 2019, RSC.

The design and construction of porous structures during the preparation of carbon materials are of great importance because of the unique properties of porous structures. Compared with nonporous carbon, porous carbon materials usually have a larger SSA, higher porosity, and lower density.^[^ ^199^ ^]^ These features are beneficial for applications such as energy storage and conversion, electrocatalysis, and gas absorption. According to the definition given by the International Union of Pure and Applied Chemistry (IUPAC), pores in materials can be categorized into three types: micropores (pore size less than 2 nm), mesopores (pore size of 2–50 nm) and macropores (pore size more than 50 nm). Micropores and mesopores can increase the SSA of the material to a large degree and thus expose more active sites. However, the pore spacing between these two kinds of pores is relatively large, which is not conducive to the diffusion of ions or reactants. Macropores can minimize the resistance of mass transport. Therefore, compared with porous materials with uniform pore sizes, hierarchical porous materials have better performance in practical applications.^[^ ^200^ ^]^ An illustration of the three types of pores is presented in **Figure** **6**a. Direct transformation of biomass molecules into carbonaceous materials can hardly produce porous structures, especially hierarchical porous structures. Thus, pretreatment of the biomass materials is necessary before carbonization. Herein, common strategies of porous structure construction are summarized below.

#### 3.2.2 Activation Method

Activation is a process during which different kinds of gas or activators can be used to act with carbonaceous materials to create micropores, mesopores, and macropores.^[^ ^204^ ^]^ The activation method is a common strategy to create porous structures. The activation process can increase the porosity and pore volume of the carbon material, as well as enlarge the pore diameters.^[^ ^205^ ^]^ The activation process includes two steps: Carbonization and activation. During the carbonization stage, biomass precursors will be transformed into carbonaceous materials at temperatures <800 °C and in the absence of oxygen.^[^ ^206^ ^]^ At the same time, noncarbon elements will be eliminated as small molecule gases, which will also induce the formation of pores. Activation is the key morphology control step, and it is important to design the parameters of the activation process to obtain the desired carbon materials.^[^ ^207^ ^]^ According to the activators used, activation can be classified into two types: Physical activation and chemical activation.

##### Physical Activation

In the physical activation process, biomass will go through two stages. The first stage is the pyrolysis of biomass precursors in an inert gas atmosphere, which will lead to partial carbonization and the elimination of noncarbon elements.^[^ ^208^ ^]^ The second stage is the activation process in an oxidizing gas atmosphere, for example, steam, carbon dioxide or an air mixture where the oxidizing gases react with the carbon skeleton.^[^ ^209^ ^]^ The reaction mechanism in a steam atmosphere can be described as

(1) $$C\;\left(s\right)+{\;H}_{2}O\left(g\right)\to {\mathrm{CO}}_{2}\left(g\right)+{\;H}_{2}\;\left(g\right)$$

While in carbon dioxide, the mechanism is^[^ ^210^ ^]^

(2) $$C\;\left(s\right)+{\;\mathrm{CO}}_{2}\left(g\right)\to 2\mathrm{CO}\left(g\right)$$

Activation time is a critical parameter in physical activation. Zgrzebnicki et al. prepared N‐doped activated carbon derived from furfuryl alcohol through CO_2_ activation.^[^ ^201^ ^]^ Different activation times were set as 15, 60, 120, and 240 min, and the burn‐off values of the samples prepared accordingly were 1%, 20%, 73%, and 88%. The adsorption and desorption isotherms of nitrogen of the above samples are presented in Figure 6b. Apparently, the adsorbed nitrogen volumes vary vastly due to activation time. The adsorbed nitrogen volume tends to increase with increasing activation time to 120 min (AC‐73), while further prolonging the activation time leads to a lowered adsorbed nitrogen volume (AC‐88).

##### Chemical Activation

Different from physical activation, chemical activation involves the addition of activator reagents, and carbonization occurs simultaneously with activation in one step. During activation, activator reagents will insert into the interior of the carbon materials, and cross‐linking polycondensation reactions will occur between the carbon skeleton and activator reagents.^[^ ^211^ ^]^ Dehydrating reagents and oxidants are widely selected as the activator, among which KOH and ZnCl_2_ are the most common activators. For ZnCl_2_ activation, the activation mechanism is not yet clear. It is thought that during activation, dehydrating will occur when the precursors are impregnated with ZnCl_2_. This process will lead to the charring and aromatization of the carbon skeleton and the formation of the porous structure.^[^ ^212^ ^]^ The mechanism of KOH activation is relatively well developed. The reactions include:^[^ ^34^ ^]^

(3) $$C\;\left(s\right)+4\mathrm{KOH}\;\left(s\right)\to K_{2}{\mathrm{CO}}_{3}\;\left(s\right)+{\;K}_{2}O\;\left(s\right)+\;2H_{2}\;\left(g\right)$$

(4) $$C\;\left(s\right)+K_{2}O\;\left(s\right)\to 2K\;\left(s\right)+\;\mathrm{CO}\;\left(g\right)$$

(5) $$K_{2}{\mathrm{CO}}_{3}\;\left(s\right)+2C\;\left(s\right)\;\to 2K\;\left(s\right)+\;3\mathrm{CO}\;\left(g\right)$$

Other alkyl metal salts, such as KCl, are also used as activators. Our group has prepared porous carbon derived from silk fibroin by KCl activation.^[^ ^74^ ^]^ It was found that the activation of KCl can create a hierarchical porous structure with micropores, mesopores, and macropores at the nanoscale, as shown in Figure 6c.

**Table** **2** summarizes different biomass‐derived porous carbon materials via physical or chemical activation and the details of their porous structure. Compared with chemical activation, physical activation requires a longer activation time, and higher activation temperature, which can consume a large amount of energy. In addition, physical activation normally has more complicated procedures, as discussed above. However, chemical activation also faces the problem of the physical mixture of the activator reagent with precursor as well as the removal of activator residue after activation. It is noteworthy that the controlled formation of ordered porous structures is still a challenge for activation methods.

**Table 2:** A summary of BCMs prepared through activation methods

Biomass	Activator	Temperature (°C)	Activation time [h]	*S*_BET_ ^a)^ [m^2^ g^−1^]	*S*_M_/*S*_BET_ ^b)^ [%]	*V*_T_ ^c)^ [cm^3^ g^−1^]	References
Corn grains	steam	900	1	1417	42	1.020	[213]
Olive tree pruning	Steam	Carbonization:500; Activation: 910	1.75	774.18	/	0.382	[214]
Coffee husk	Steam	Carbonization:500; Activation: 910	1.75	1447.41	/	0.699	[214]
Waste Tea	Steam	Carbonization:450; Activation: 800	0.5	995.07	/	0.678	[215]
Spent coffee grounds	Steam	Carbonization:450; Activation: 800	1	981.12	24.9	1.03	[216]
Peanuts	CO_2_	Carbonization:600; Activation: 850	2	1817	72.7	0.880	[217]
Hybrid willow biomass	CO_2_	800	1	738.74	68.7	0.37	[218]
Hemp fibers	CO_2_	Carbonization:600; Activation: 850	30	1060	/	0.1	[219]
Peanuts	KOH	700	1	2420	49.8	1.117	[217]
Olive tree pruning	KOH	Carbonization:500; Activation: 910	1	2662.15	/	1.107	[214]
Coffee husk	KOH	Carbonization:500; Activation: 910	1	2275.71	/	0.857	[214]
Spent coffee grounds	KOH	850	0.5	2265.10	97.2	1.17	[216]
Olive tree pruning	K_2_CO_3_	800	1	1477.42	/	0.604	[214]
Coffee husk	K_2_CO_3_	800	1	1156.51	/	0.379	[214]
Pomelo peels	ZnCl_2_	500	2	1361	60.9	1.57	[48]
Lotus pollens	CuCl_2_	800	2	1722.5	91.7	0.95	[220]

^a)^ Specific surface area determined by the Brunauer‒Emmett‒Teller (BET) method

^b)^ The ratio of the specific surface area of micropores to the total specific surface area

^c)^ Total pore volume.

#### 3.2.3 Template Method

The template method uses templates to construct porous structures, during which templates are impregnated into biomass precursors. After carbonization and the removal of templates by a strong acid or strong base, pores with uniform sizes will be created.^[^ ^221^ ^]^ The most significant advantage of this method is its ability to prepare ordered porous morphology according to various templates, which cannot be achieved by activation. According to the features of templates, template methods can be categorized as either soft templates or hard templates.

##### Soft Templates

Soft templates are organic molecules that can form a framework when they interact with biomass precursors. Soft templates include surfactants, block copolymers, and ionic micelles.^[^ ^222^ ^]^ These molecules will self‐assemble into micelles or vesicles when the concentration exceeds the critical concentration and interact with carbon precursors via hydrogen bonds, hydrophobic, or hydrophilic actions together with the electrostatic interactions to form a coat on the precursor.^[^ ^34^ ^]^ During carbonization, these templates will be decomposed, and thus, a porous structure will remain in the obtained carbon materials.^[^ ^223^ ^]^ Nelson et al. chose a triblock copolymer, Pluronic F127 (EO_106_PO_70_EO_106_, 12 600 kDa), as a soft template and prepared mesoporous carbons derived from chestnut tannin, as shown in Figure 6d.^[^ ^202^ ^]^ The feasibility of the porous structure construction of chestnut tannin via the Pluronic F127 soft template can be attributed to the presence of gallic acid, which is key to the interaction between the triblock copolymer and phenolic carbon precursor of tannin through hydrogen bonding. Due to the poor thermal stability of template molecules, hydrothermal reactions at relatively low temperatures are a common strategy for the formation of template frameworks, as summarized in **Table** **3**.

**Table 3:** A summary of BCMs prepared through the soft template method

Biomass	Template	Template synthesis temperature [°C]	Template removal Temperature [°C]	*S*_BET_ [m^2^ g^−1^]	*S*_M_/*S*_BET_ [%]	*V*_T_ [cm^3^ g^−1^]	References
Chestnut tannin	F127	80	2	420	66.2	0.43	[202]
Glucosamine	P123	180	600	980	53.0	0.78	[224]
Lignin	P123	180	600	371	/	0.30	[225]
Chitosan	F127	220	800	326	61.6	0.20	[226]
Chitosan	IL	220	800	363	0.67	0.21	[226]
Batatas	F127	160	800	344.4	71.3	0.17	[227]
Walnut shell	F127	100	600	537	/	0.31	[228]
Gallic acid	F127	100	600	538.6	/	/	[229]

##### Hard Templates

Compared with the soft template method, the hard template method is easier. The principle of the hard template method is to fill monomer templates into biomass precursors by physical or chemical methods, and porous carbons can be obtained after carbonization and the removal of templates. Typical hard templates include MgO,^[^ ^230^ ^]^ ZnO,^[^ ^231^ ^]^ and SiO_2_,^[^ ^232^ ^]^ among which SiO_2_ is the most commonly used template. The porous morphology of the carbon materials can be tuned according to the selected template. Li et al. used a novel composite hard template of Mg_5_(OH)_2_(CO_3_)_4_/ZnCl_2_ followed by HCl etching to prepare hierarchically structured porous carbon materials derived from glucose/urea.^[^ ^203^ ^]^ The template was mixed with biomass by physical grinding. The hierarchical porous structure can be attributed to the synergetic effect of grinding, templating and acid etching, as shown in Figure 6e. For the porous construction mechanism of Mg_5_(OH)_2_(CO_3_)_4_/ZnCl_2_ templates, ZnCl_2_ is converted into ZnO with increasing temperature and then reduced to Zn due to the formation of a carbon matrix. Zn sublimated from the in situ‐formed carbon matrix as vapor, which created meso‐ and macropores. Mg_5_(OH)_2_(CO_3_)_4_ would become MgO during carbonization, and mesopores were formed after acid etching. Figure 6f is an illustration of this process. Moreover, this dual‐templating method was demonstrated to be applicable to other biomass precursors, such as roots, stems, leaves, flowers, and fruits of various plants. Different from soft templates, hard templates can hardly decompose during calcination. Therefore, post treatment to remove the template residue, such as acid and water rinse, is always necessary. Applications of various templates and the specific details of the porous structure of the obtained carbon materials are summarized in **Table** **4**.

**Table 4:** A summary of BCMs prepared through the hard template method

Biomass	Template	Calcination temperature [°C]	Template removal	*S*_BET_ [m^2^ g^−1^]	*S*_M_/*S*_BET_ [%]	*V*_T_ [cm^3^ g^−1^]	References
Soybean milk powder	CaCO_3_	700	1 m HCl and water washing	1208	81.7	0.7	[233]
Cornstalk	CaCO_3_	800	1 m HCl solution and water washing	2054	/	1.382	[234]
Pyrolysis oil	ZnO (20 nm) particles	900	3 m HCl and water washing	1770	5.1	/	[235]
Almonds	Poly‐methyl methacrylate (PMMA)	800	/	1877.8	/	0.67	[236]
Heavy fraction of bio‐oil	Crayfish shell	400	Excess HCl	3095	/	1.66	[237]
Glucose	SiO_2_	700	2 m NaOH solution and water rinse	237.33	/	0.33	[238]
Waste gelatin	SBA‐15 (highly ordered hexagonal mesoporous SiO_2_)	800	10 wt% HF washing	818.3	/	1.05	[239]
Rice starch	SBA15 (SiO_2_ Nano‐Template)	900	5 wt% HF washing	915	/	1.12	[240]
Chitosan	AlCl_3_·6H_2_O	700	6 wt% HCl aqueous solution and water washing	554.1	61.3	0.747	[241]
Lotus seed shell	nano‐Na_2_CO_3_ and nano‐Na_3_PO_4_ particles	650	HCl washing	3188	/	3.20	[242]

## 4 Synthesis of Biomass‐Derived Carbon Materials

**Figure 7:**
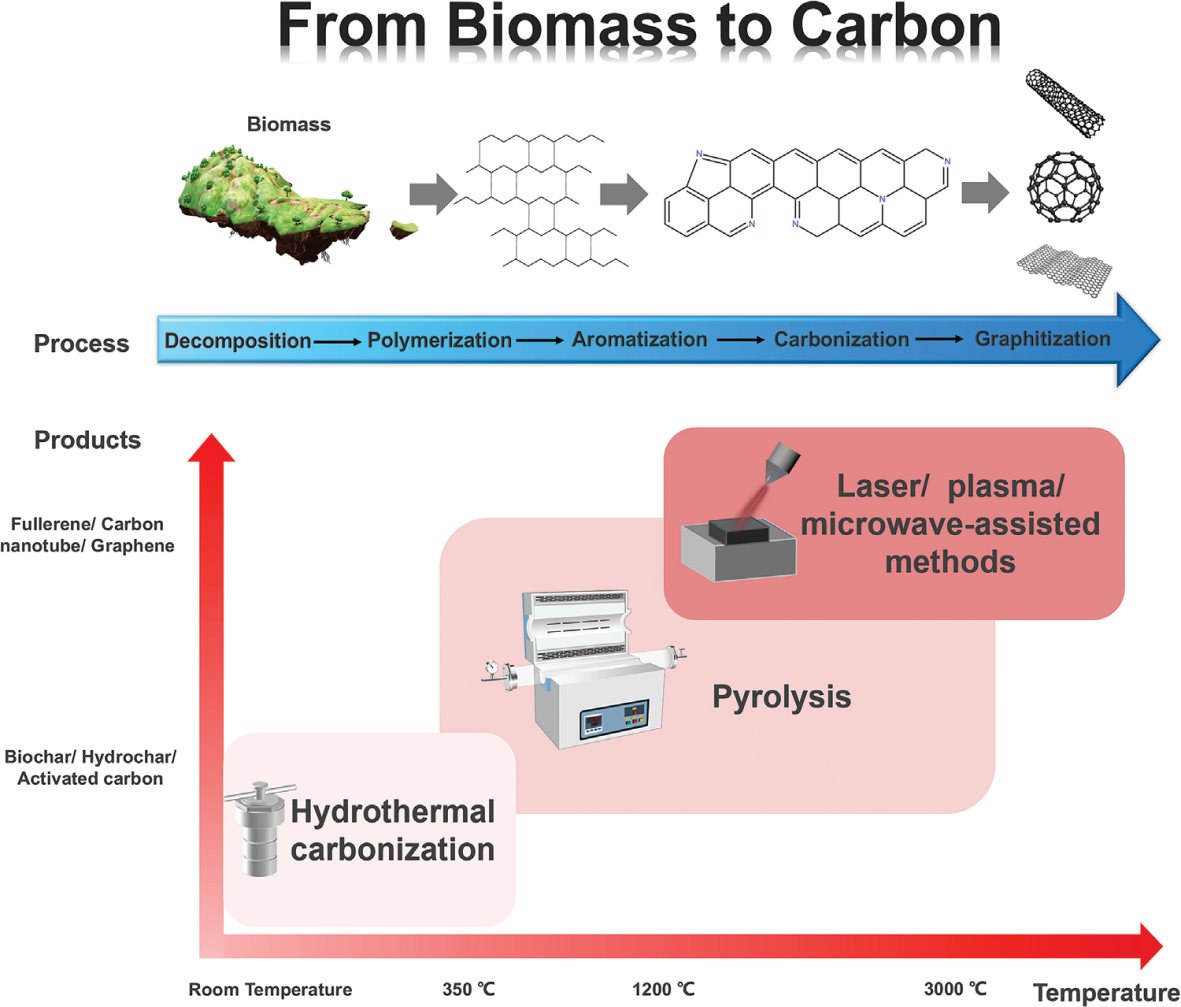
A summary of the transformation process from biomass precursors to carbonaceous materials and typical preparation methods. Top: The transformation process from biomass precursors to carbon. Bottom: Different preparation methods and the working temperature as well as typical carbon products of each methods.

Although biomass precursors have various molecular structures, the common conversion stages during the preparation of BCMs include decomposition, polymerization, aromatization, carbonization, and graphitization,^[^ ^243^ ^]^ as illustrated in **Figure** **7**. It is noteworthy that the existence of metal elements and other hetero elements is unavoidable in biomass precursors. However, the metal content in the precursor is relatively low and can be removed by acid treatment after carbonization.^[^ ^34^ ^]^ Heteroatoms can be introduced in situ in the carbon matrix by direct carbonization to obtain heteroatom‐doped carbons, and they can be eliminated by high carbonization temperatures. To obtain carbonaceous materials with different structures, many approaches have been exploited, such as HTC, pyrolysis, and other methods, including laser/plasma/microwave‐assisted methods, which are summarized in Figure 7. In this chapter, the carbonization process of different biomass precursors will be discussed, and typical synthesis strategies of BCMs will be introduced.

### 4.1 Carbonization Process of Biomass

#### 4.1.1 Carbonization Process of Plant Biomass

**Figure 8:**
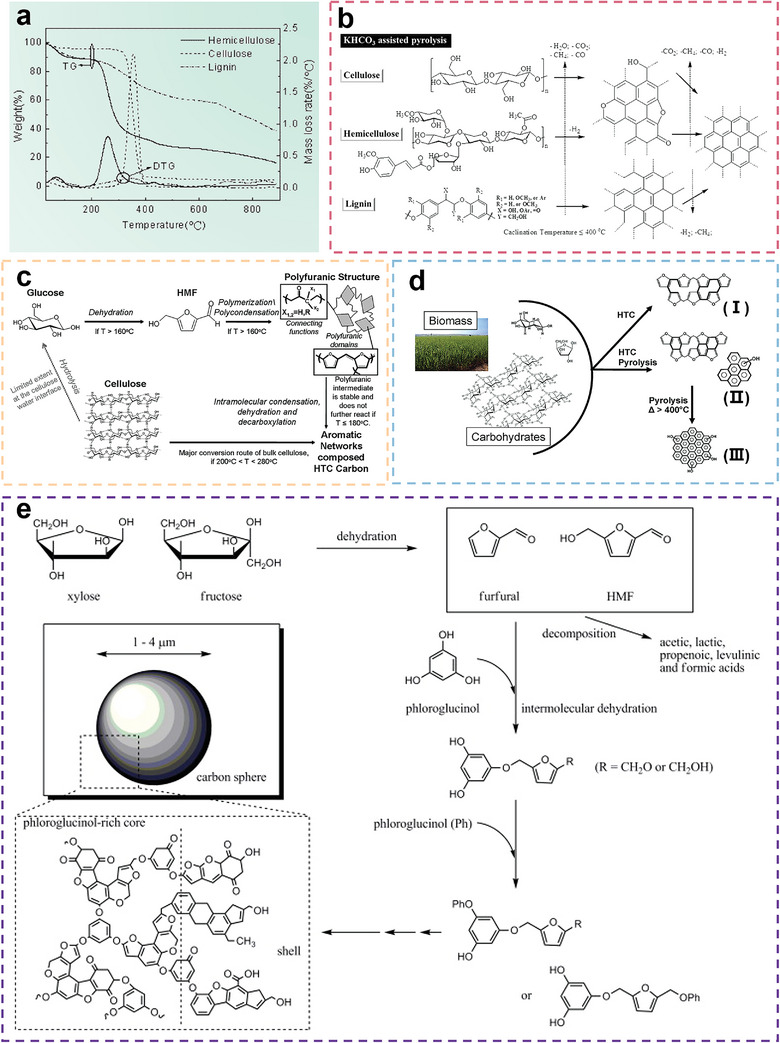
a) Pyrolysis curves of cellulose, hemicellulose, and lignin. Reproduced with permission.^[^ ^245^ ^]^ Copyright 2006, ACS. b) Possible structure transformation process into carbon of cellulose, hemicellulose, and lignin during KHCO_3_‐assisted pyrolysis. Reproduced with permission.^[^ ^246^ ^]^ Copyright 2016, ACS. c) Cellulose derived carbon formation mechanism via HTC under mild processing conditions (180 °C < *T* < 280 °C). Reproduced with permission.^[^ ^247^ ^]^ Copyright 2011, RSC. d) Possible structures of carbohydrate biomass during hydrothermal or pyrolysis. Reproduced with permission.^[^ ^248^ ^]^ Copyright 2011, ACS. e) Carbon sphere formation mechanisms of monosaccharides via HTC. Reproduced with permission.^[^ ^249^ ^]^ Copyright 2010, Elsevier.

For the carbonization of plant biomass, both pyrolysis and hydrothermal approaches are commonly used. With the help of thermogravimetry analysis (TGA) and gas chromatography (GC)/mass spectrometry (MS), the carbonization behavior of plant biomass is well developed.^[^ ^244^ ^]^ Yang et al. investigated the pyrolysis of cellulose, lignin and hemicellulose.^[^ ^245^ ^]^ **Figure** **8**a shows the pyrolysis curves of cellulose, lignin and hemicellulose. The decomposition of cellulose initiates at 315 °C, and the mass‐loss rate reaches a maximum of 2.1 wt% °C^−1^ at 355 °C. The solid residue of cellulose after 900 °C is 7%. Hemicellulose has the lowest decomposition temperature, which is only 220 °C, but the mass remaining is 20%, which is higher than that of cellulose. Lignin has a quite different pyrolysis behavior, the mass‐loss rate was stable, and there was no sharp decrease in the TGA curves. Lignin also has the largest residue remaining at 40%. The difference in the pyrolysis process can be attributed to the molecular structure. For hemicellulose, the molecule is amorphous and has little strength, while the molecule of cellulose is a long polymer chain without many branches and is crystalline. The lignin molecule consists of three types of benzene‐propane, and thus, the molecule is heavily cross‐linked, indicating high thermal stability.

Deng et al. probed the revolutionary route of cellulose, hemicellulose, and lignin to carbon structures under KHCO_3_‐assisted pyrolysis, as shown in Figure 8b.^[^ ^246^ ^]^ The hydroxyl groups in cellulose and hemicellulose can be primarily transformed into oxygen heterocyclic rings and further into aromatic rings. In addition, hydroxyl groups of cellulose or hemicellulose can be connected to form 3D structures due to dehydration and condensation during pyrolysis. However, for lignin, this process can be quite different. During pyrolysis, a series of condensation and addition reactions are triggered under heat, and aromatics are formed in a 2D manner.

Falco et al. investigated the carbonization process of cellulose via HTC under mild processing conditions (180 °C < *T* < 280 °C).^[^ ^247^ ^]^ The possible structural transformation process is shown in Figure 8c. When the hydrothermal temperature is above 180 °C, the fibril structure of cellulose will be destroyed and broken into several fragments at the nano‐ or microscales. These broken pieces can form a spherical envelope that will protect internal cellulose from the surrounding water interface, thus reducing the possibility of hydrolysis of the glycosidic bond. Therefore, most of the cellulose inside the envelope structure is likely under a thermal environment similar to pyrolysis. Intramolecular condensation, dehydration, and decarboxylation reactions will occur under this condition, and the obtained carbon structure is composed of aromatic networks. Moreover, cellulose on the envelope‐water interface undergoes hydrolysis to form glucose simultaneously due to contact with water. It is noteworthy that the cellulose‐glucose transformation process only accounts for a limited part of the whole reaction mechanism. They also probed the different structures of carbohydrate‐derived carbons during hydrothermal and pyrolysis processes.^[^ ^248^ ^]^ As shown in Figure 8d, structure I is a furan‐rich structure that can only be observed in the hydrothermal process. This structure is also temperature‐, time‐, and source‐dependent and appears either at low residence times or at HTC temperatures below 200 °C. Structure II exists under both hydrothermal and pyrolysis treatments. The feature of this structure is large amounts of arene groups produced either by condensed three‐membered furanic units or by polycyclic aromatic hydrocarbon clusters. Structure III can be found under high calcination temperatures, where the number of arene units increases while the amount of both furanic and phenolic groups decreases.

Ryu et al. proposed the microsphere formation mechanism during HTC of monosaccharides (xylose for pentose and fructose for hexose) with phenolic compounds, as shown in Figure 8e.^[^ ^249^ ^]^ The carbonization process begins with the dehydration of monosaccharides. They are transformed into furan compounds, furfural for xylose and 5‐(hydroxymethyl)‐2‐furaldehyde (HMF) for fructose. These furan products then react with phloroglucinol through intermolecular dehydration, resulting in the formation of soluble polymers. Under some circumstances, the furan products will self‐decompose into organic acids, such as acetic and formic acid. In addition, the hydronium ions from these acidic products can act as catalysts in the follow‐up stages.^[^ ^250^ ^]^ In the next stage, soluble polymers will aromatize via keto‐enol tautomerization or intramolecular dehydration to form C = C bonds.^[^ ^251^ ^]^ Then, the aromatized cluster nucleate and the formed nuclei grow with the assistance of the diffusion and linkage of the chemical species with reactive oxygen surface functionalities in the solution. It is noteworthy that at the initial stage of the nuclei growth, phloroglucinol, rather than the furans or acids, act as the cross‐linker which leads to their concentration at the core of the carbon sphere. Reactive oxygen groups, including hydroxyl, carbonyl, carboxylic and ester, as well as, C—OH, C—O—C bonds tend to locate at the surface of the carbon sphere. Inada et al. analyzed the structural evolution from glucose to carbon spheres via HTC and further to graphite carbon by heat treatment.^[^ ^252^ ^]^ Similar to the HTC process of fructose, glucose will first be dehydrated to HMF. Then, HMF decomposes to levulinic acid and formic acid by hydrolysis.^[^ ^253^ ^]^ The formed acids can release protons until the system reaches the equilibrium and the released protons accelerate the formation of HMF, leading to the accumulation of HMF.^[^ ^254^ ^]^ Afterward, the accumulated HMF polymerizes to form an oil/water emulsion. During this process, the long chain of levulinic acid acts as the emulsifier.^[^ ^255^ ^]^ The polymerization of HMF occurs in the oil droplet, leading to the formation of carbon spheres. At this stage, graphite structure can be found in the carbon spheres, but it is mainly comprised of polymerized HMF with a stacking structure. By heat treatment of carbon spheres, cross‐linking of disorganized carbon and microcrystalline graphite occur and poorly graphitized carbon is formed. Simsir et al. investigated the nuclear magnetic resonance spectra of the glucose hydrochar and glucose raw material.^[^ ^256^ ^]^ The peaks attributed to sp^3^ hybridized carbons belonging to the aliphatic region, sp^2^ hybridized carbons, and carbonyl functional groups can be found in the spectra of the hydrochar.

#### 4.1.2 Carbonization Process of Animal Biomass

**Figure 9:**
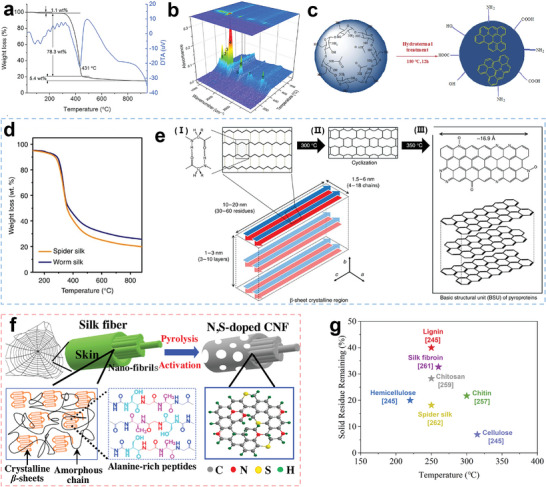
a) TGA and DTA curves of chitin. Reproduced with permission.^[^ ^257^ ^]^ Copyright 2015, WILEY‐VCH. b) 3D on‐line FTIR spectra of chitin during carbonization, and the labelled peaks are 1) C—O stretching, 2) O—H bending, 3) N—H bending, 4) C = O stretching, 5) CO, 6) CO_2_, 7) C—H stretching, and 8) O—H stretching. Reproduced with permission.^[^ ^257^ ^]^ Copyright 2015, WILEY‐VCH. c) Increase in the surface functional groups of chitosan after HTC treatment. Reproduced with permission.^[^ ^260^ ^]^ Copyright 2015, WILEY‐VCH. d) TGA curves of worm silk and spider silk. Reproduced with permission.^[^ ^262^ ^]^ Copyright 2015, The authors, Springer Nature. e) Schematic of the formation of BSUs. Reproduced with permission.^[^ ^262^ ^]^ Copyright 2015, The authors, Springer Nature. f) Schematic of the preparation of spider silk‐derived carbon fiber. Reproduced with permission.^[^ ^263^ ^]^ Copyright 2016, Elsevier. g) Decomposition temperature and solid residue remaining in several plant and animal biomasses.

Animal biomasses, such as chitin, chitosan and protein, have complicated carbonization processes. The decomposition of chitin starts at 300 °C, and the weight loss is as high as 78.3 wt% after carbonization according to Gao et al.^[^ ^257^ ^]^ **Figure** **9**a shows the TGA and differential thermal analysis curves of chitin. According to the 3D on‐line FTIR spectra of chitin during carbonization shown in Figure 9b, the weight loss occurring in the period of 300–450 °C can be attributed to the degradation of polysaccharides. Chitin will be deacetylated, and the deacetylated pyranose rings will be decomposed during this process. Moreover, some nitrogen will be eliminated in the form of nitrogen oxide in this stage. They also found that the nitrogen content in the prepared carbon decreases with increasing carbonization temperature. Nogi et al. prepared nanofibrillar carbon from chitin nanofibers.^[^ ^258^ ^]^ The carbon content in chitin‐derived carbon increases to 83.1% from 44.7%, and the nitrogen content also increases slightly from 6.2% to 8.1%, due to the nitrogen atoms of the acetamide groups remaining after heat treatment. The decomposition temperature of chitosan is 250 °C, which is lower than that of chitin, and the solid residue remaining is ≈28%.^[^ ^259^ ^]^ Rafiee et al. used a hydrothermal method to synthesize chitosan‐derived carbon quantum dots.^[^ ^260^ ^]^ The hydrothermal treatment of chitosan can increase the number of functional groups on the surface, such as amino, hydroxyl, and carboxyl groups (Figure 9c).

Silk is a typical protein biomass widely used to prepare BCMs. Silk fibroin is the main component of silk fiber. During pyrolysis, silk fibroin decomposes when the temperature exceeds 260 °C, and after 800 °C carbonization, only 32.7% of silk fibroin remains.^[^ ^261^ ^]^ Cho et al. investigated the carbonization process of silk protein and the molecular transformation from fibroin into pseudographitic pyroprotein.^[^ ^262^ ^]^ Figure 9d shows the TGA curves of worm silk and spider silk. It can be observed that a rapid weight loss occurs in the temperature period of 250–300 °C, which is due to the thermal decomposition of the protein backbone in the amorphous region. The molecular transformation process of the *β*‐sheet structure of silk fibroin during pyrolysis is illustrated in Figure 9e. Figure 9e‐I shows the *β*‐sheet structure of silk fibroin, which is composed of two (or more) protein chains, and the structure is stabilized by many hydrogen bonds formed between the amide and carbonyl oxygen of adjacent peptide chains. When the temperature is over 300 °C, intermolecular dehydration or condensation between adjacent molecular chains may occur, which leads to cyclization or aromatization of silk fibroin molecules (II in Figure 9e); thus, a heteroaromatic BSU is formed (III in Figure 9e). In addition, a *β*‐sheet structure consisting of peptides with small side chains, for example glycine or alanine, can induce intersheet stacking which leads to the formation of a 3D structure because of van der Waals interactions, thus resulting in the appearance of the (002) plane of stacked carbon.

The structure of spider silk is similar to that of worm silk, which is composed of a highly oriented alanine‐rich *β*‐sheet structure and amorphous glycine‐rich peptide chains. Therefore, they exhibit similar pyrolysis behavior. Zhou et al. took advantage of the high N content feature of spider silk and prepared N, S‐doped carbon fiber from spider silk, as shown in Figure 9f.^[^ ^263^ ^]^ The N content after 700 °C carbonization is up to 4.1%. The decomposition temperature and solid residue remaining in several biomass precursors are summarized in Figure 9g.

### 4.2 Synthesis Methods of Biomass‐Derived Carbon Materials

#### 4.2.1 Hydrothermal Carbonization

**Figure 10:**
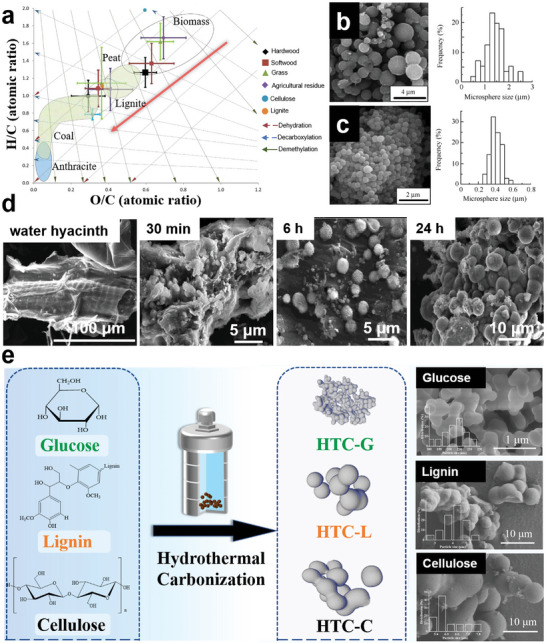
a) Delineation of the reaction pathway of hydrochar from various feedstock shown by Van Krevelen Diagram. Reproduced with permission.^[^ ^264^ ^]^ Copyright 2020, Elsevier. b) SEM and size histogram of glucose hydrochar carbonized at 170 °C. Reproduced with permission.^[^ ^270^ ^]^ Copyright 2009, WILEY‐VCH. c) SEM and size histogram of glucose hydrochar carbonized at 230 °C. Reproduced with permission.^[^ ^270^ ^]^ Copyright 2009, WILEY‐VCH. d) SEM images of water hyacinth and hydrochar processed under different residence time. Reproduced with permission.^[^ ^271^ ^]^ Copyright 2013, Elsevier. e) Preparation of glucose‐, lignin‐, and cellulose‐derived carbons and their morphologies. Reproduced with permission.^[^ ^272^ ^]^ Copyright 2021, Elsevier.

HTC has been widely adopted to prepare carbonaceous materials from high moisture content biomass, such as cellulose, lignin, and hemicellulose.^[^ ^264^ ^]^ It has been considered a promising way to transfer waste biomass to high‐value carbon materials. The principle of HTC is to simulate the natural coalification process in a short time by the coeffect of high temperature and pressure. Biomass precursors are mixed with water at an appropriate ratio and are sealed in an HTC reactor for thermal treatment at temperatures between 180 and 350 °C for a certain residence time.^[^ ^265^ ^]^ Several reactions, such as condensation, polymerization, hydrolysis, decarboxylation, dehydration, and aromatization, occur during HTC. Although the exact reaction route of biomass during HTC is not yet fully understood, hydrolysis where ester and other bonds breakdown into fragments, among all the reactions occurring, is considered the governor of HTC due to its low activation energy.^[^ ^266^ ^]^ The H/C and O/C ratios are reduced by dehydration and decarboxylation,^[^ ^267^ ^]^ as shown in the Van Krevelen diagram (**Figure** **10**a). During the dehydration process, hydroxyl groups (—OH) are eliminated, which improves the carbonization rates and significantly reduces the O/C ratio.^[^ ^264^ ^]^ When HTC temperature is above 150 °C, carboxyl (—COOH) groups are rapidly eliminated, producing mostly CO_2_ and CO.^[^ ^268^ ^]^ Condensation and polymerization can lead to the participation of small active fragments produced from previous reactions. The aromatic building blocks of the carbonaceous material are obtained during aromatization reactions.^[^ ^268^ ^]^ However, the orders and interactions of these reactions are not yet clear.^[^ ^269^ ^]^

For the preparation of BCMs via the HTC method, parameters such as HTC temperature, time of residence, use of catalysts and water biomass ratio, have a large effect on the structure of the obtained carbons and should be carefully designed. Zhang et al. studied the effect of HTC conditions on the characteristics of hydrochar pellets derived from wheat straw.^[^ ^273^ ^]^ It is found that the HTC temperature has a significant influence on the structure of hydrochar. With increasing temperature (from 180 to 260 °C), the oxygen content in the hydrochar decreases to a large degree (from 42.46% to 23.41%). Sevilla et al. found that the HTC temperature leads to a difference in the diameters of the carbon spheres derived from glucose.^[^ ^270^ ^]^ The diameter of the carbon spheres obtained at an HTC temperature of 170 °C is 0.4 ± 0.06 µm, while that of the samples treated at 230 °C is 1.4 ± 0.4 µm. Figure 10b,c show scanning electron microscopy (SEM) images and size histograms of glucose hydrochar carbonized at 170 °C and 230 °C, respectively.

The residence time of HTC is another important factor that can lead to structural differences. Gao et al. investigated the effect of residence time on the morphology of water hyacinth derived carbon.^[^ ^271^ ^]^ The results suggest that a short residence time will result in cracks and trenches while a longer residence time (larger than 6 h) will induce the formation of microspheres or carbon microspheres, which can be ascribed to the complete decomposition of the raw materials.^[^ ^271^ ^]^ The morphology images of water hyacinth and hydrochar obtained under different residence times are presented in Figure 10d. Romero‐Anaya et al. chose glucose and sucrose as hydrochar precursors and studied the residence time effect on morphology.^[^ ^274^ ^]^ It is found that carbon spheres with well‐defined morphology were obtained at longer residence time.^[^ ^274^ ^]^ HTC residence time will also affect the porosity of BCMs and longer residence time is beneficial to the formation of defined structure porosity, pore volume as well as high SSA.^[^ ^275^ ^]^ Previous study by He et al. showed that the fragmentation and porosity of hydrochars derived from dried sewage sludge increased with the increasement of HTC residence time which is due to the release of volatile gases during devolatilization and the break‐down of chemical bonds of the sludge matrix.^[^ ^276^ ^]^

Due to the different thermal decomposition behavior of biomass components, the effect of biomass feedstock on the structure of BCMs via HTC method is not negligible. Normally, biomass feedstock with higher lignin content produces more biochar than hemicellulose‐rich or cellulose‐rich feedstock because of the hardness of degradation of lignin.^[^ ^277^, ^278^ ^]^ Chen et al. prepared hydrothermal carbon from glucose, lignin and cellulose, as illustrated in Figure 10e.^[^ ^272^ ^]^ The carbon materials they prepared by hydrothermal treatment exhibit similar spherical morphologies of precursor particles, but lignin‐ and cellulose‐derived hydrothermal carbons have larger average sizes than their precursor particles.

The use of additives in HTC will change the HTC environment and thus has unique advantages. The addition of LiCl can reduce the pressure during HTC, which improves the safety of preparation and lowers the cost of HTC due to the use of less expensive reactor vessels.^[^ ^279^ ^]^

#### 4.2.2 Pyrolysis Method

Carbonization through pyrolysis is another effective and feasible strategy to synthesize BCMs. During pyrolysis, biomass is carbonized under a certain atmosphere (nitrogen, argon, etc.) at high temperature in a tubular furnace. The properties of BCMs can be tailored by controlling the carbonization parameters, such as temperature and heating rate.^[^ ^280^ ^]^

**Figure 11:**
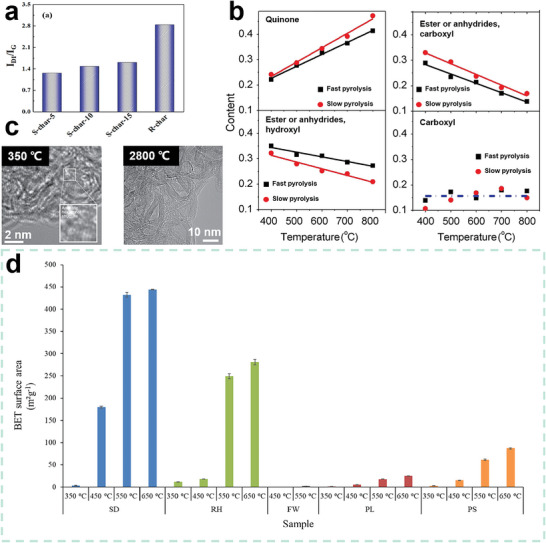
a) *I*_D_/*I*_G_ values obtained from the curve‐fitted Raman spectra of Arundo donax‐derived carbon; the numbers 5, 10, and 15 refer to the heating rate. Reproduced with permission.^[^ ^282^ ^]^ Copyright, 2020 Elsevier. b) Content of the XPS peaks of walnut shell‐derived carbon. Reproduced with permission.^[^ ^283^ ^]^ Copyright 2019, Elsevier. c) HR‐TEM images of the silk protein‐derived carbon heated to 350 and 2800 °C. Reproduced with permission.^[^ ^262^ ^]^ Copyright 2015, The authors, Springer Nature. d) BET surface area of different BCMs. Reproduced with permission.^[^ ^285^ ^]^ Copyright 2020, Elsevier.

The heating rate is one of the key operating parameters that has a great impact on the BCM structure. Studies have pointed out that a slow pyrolysis process can lead to a highly ordered carbon structure.^[^ ^281^ ^]^ Liu et al. investigated the structural features of Arundo donax‐derived carbon via various heating rates.^[^ ^282^ ^]^ Samples prepared by a fast heating rate have more structural defects and crystalline imperfections with larger values of *I*_D_/*I*_G_ calculated from Raman spectra (**Figure** **11**a). In addition, Arundo donax‐derived carbons from a slow heating rate possess a higher crystallization size, graphitization and aromatization degree than fast pyrolysis products. Yuan et al. compared BCMs from walnut shells through fast and slow pyrolysis.^[^ ^283^ ^]^ It was found that the slow pyrolysis process produces more free radical fragments than fast pyrolysis. Figure 11b shows the content of the peaks of different functional groups obtained from X‐ray photoelectron spectroscopy (XPS) spectra. The contents of quinine, ester and anhydride groups in carbons obtained from slow pyrolysis are larger than those of fast pyrolysis under the same carbonization temperature, while the content of hydroxyl groups is opposite.

Carbonization temperature is another significant preparation condition, affecting both element composition and carbon structure. A conjugated system of silk fibroin treated at 350 °C can be observed by high‐resolution transmission electron microscopy with hexagonal aromatic rings with dimensions of ≈2.5 Å (Figure 11c). When the pyrolysis temperature increased to 2800 °C, a highly developed graphitic structure was formed, as shown in Figure 11c.^[^ ^262^ ^]^ The heteroatom contents in the BCM will decrease as the carbonization temperature increases. For example, the O content in buckwheat husk‐derived carbon decreases from 5.79% to 3.97% when the pyrolysis temperature increases from 700 to 1300 °C.^[^ ^284^ ^]^ It is noteworthy that the content ratio of C = O bonds in oxygen‐containing functional groups increases with increasing temperature. Pariyar et al. found that the pyrolysis temperature also influences the SSA of BCMs after comparing BCMs derived from five feedstocks of pine saw dust (PD), rice husk (RH), food waste (FW), poultry litter (PL), and paper sludge (PS) at different pyrolysis temperatures (350, 450, 550, and 650 °C).^[^ ^285^ ^]^ As shown in Figure 11d, the SSA of the five types of BCMs increases with increasing operating temperature.

Hold time during the pyrolysis method is of great importance, especially for the preparation of activated BCMs. Lua studied the effect of the hold time during pyrolysis on the pore characteristics of oil palm shell‐derived carbons by steam activation.^[^ ^286^ ^]^ In total, a pyrolysis time of 2 h produces the best porous structure with the largest BET surface area, micropore surface area, and total pore volume. It is speculated that most of the low‐ and high‐molecular‐weight volatiles were released when the hold time is 2 h, while slight shrinkage of the pore structure in the carbon may occur if the hold time exceeds 2 h.

The atmosphere in the furnace can be adjusted from inert gas to active gas, which can be an approach for atom doping in BCMs. Li et al. carbonized bamboo under an NH_3_ atmosphere for N doping.^[^ ^287^ ^]^ They also investigated the interaction between activation and ammonia modification. The results indicate that the one‐step method, which is the activation in an NH_3_ atmosphere, is more beneficial to both N‐doping and pore characteristics. In addition, pyrolysis under active gas can induce a special structure of BCMS. Gao et al. carbonized cellulose paper under an O_2_—NH_3_ atmosphere, where cellulose chains can be spatially separated and carbonized due to preoxidation and aminolysis.^[^ ^41^ ^]^ Therefore, the carbon paper exhibits a 3D network composed of graphitic sheets.

#### 4.2.3 Laser Induced Graphitization

**Figure 12:**
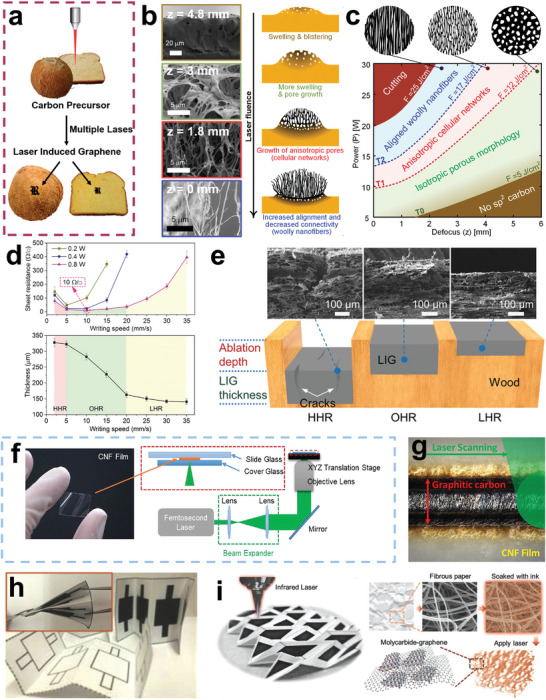
a) Schematic of BCMs produced by LIG. Reproduced with permission.^[^ ^299^ ^]^ Copyright 2018, ACS. b) Schematic of morphology evolution with the increase of laser fluence. Reproduced with permission.^[^ ^290^ ^]^ Copyright 2021, ACS. c) Morphology diagram mapping the ranges of laser parameters for producing different types of laser induced carbon materials using a continuous‐wave CO_2_ laser (wavelength *λ* = 10.5 mm) at 500 mm s^−1^. Reproduced with permission.^[^ ^290^ ^]^ Copyright 2021, ACS. d) Line charts depicting the relationship between writing speed and sheet resistance (top) and LIG produced graphene thickness (bottom). Reproduced with permission.^[^ ^293^ ^]^ Copyright 2019, WILEY‐VCH. e) Schematic illustration of the changes of graphene formation on wood by varying the writing speed of femtosecond laser. Reproduced with permission.^[^ ^293^ ^]^ Copyright 2019, WILEY‐VCH. f) Preparation setup of CNF‐derived carbon. Reproduced with permission.^[^ ^297^ ^]^ Copyright 2021, ACS. g) Morphology of CNF‐derived carbon by LIG. Reproduced with permission.^[^ ^297^ ^]^ Copyright 2021, ACS. h) Photograph of the foldable electrodes on paper substrates. Reproduced with permission.^[^ ^298^ ^]^ Copyright 2018, WILEY‐VCH. i) LIG preparation process of MCG (black color areas) on a paper substrate (white color areas) and the conversion process of paper substrates. Reproduced with permission.^[^ ^298^ ^]^ Copyright 2018, WILEY‐VCH.

Laser induced graphitization (LIG) is an attractive strategy to convert biomass into carbon materials. Different from HTC and pyrolysis methods, LIG can carbonize the selective area of the biomass and produce conductive graphitic carbon, as shown in **Figure** **12**a. The area where the laser is irradiated will undergo a series of optical and thermal reactions, such as photolysis and pyrolysis. Thus, LIG is a promising method to prepare electronic devices from biomass.

The morphology and electrical properties of LIG‐produced BCMs can be tailored by adjusting the manufacturing parameters.^[^ ^288^ ^]^ One of the key preparation conditions is the laser radiation energy. Duy et al. probed the parameters required for LIG and found that an increase in laser radiation energy leads to the formation of graphitic carbon in a fluid dynamic manner, during which the morphology changes from initial carbon sheets to fibers and eventually to droplets.^[^ ^289^ ^]^ Laser fluence value is another parameter that will affect the morphology of LIG products according to the systematic research by Abdulhafez et al.^[^ ^290^ ^]^ The continuous change of fluence change was achieved by patterning individual lines with a 10.6 µm CO_2_ laser at 18.4 W and 500 mm s^−1^ on a tilted polyimide substrate to tune the defocusing level (z). The average laser fluence increases with the decrease of *z* from 4.8 to 0 mm. Some swelling can be observed at *z* = 4.8 mm while defined porous structure with anisotropic pores emerged with less defocusing level (i.e., *z* = 3 mm). At *z* = 1.8 mm, alignment in a highly anisotropic cellular network structure appears. The formation of nanofibers initiates at *z* = 1.2 mm, and more voluminous woolly morphology formed when defocusing level approaches 0 mm. The morphology evolution of laser‐induced nanocarbon at different defocusing levels is presented in Figure 12b. Using this strategy, Abdulhafez et al also concluded the relationship between resulting morphologies and accessible laser processing parameters, such as power and defocus, as shown in the diagram of Figure 12c.^[^ ^290^ ^]^

The electrical conductivity of LIG‐produced carbon materials can be easily controlled over a wide range by tuning the laser power and writing speed.^[^ ^291^ ^]^ Lin et al. probed the influence of laser power on the sheet resistance of 3D porous graphene films produced by LIG.^[^ ^292^ ^]^ The sheet resistance of the graphene thin film decreased from 35 to 15 35 Ω sq^−1^ with the increase of the threshold power from 2.4 to 5.4 W. Le et al. found that the writing speed of the laser will alter the sheet resistance of wood derived carbon by LIG.^[^ ^293^ ^]^ The sheet resistance curves along with different writing speeds exhibit a U‐shape with three regions: high heat‐accumulation region (<5 mm s^−1^), optimized heat‐accumulation region (OHR; 5–20 mm s^−1^), and low heat‐accumulation region (>20 mm s^−1^), as shown in the upper line chart of Figure 12d. In HRR region, long exposure time leads to the increase of the surface temperature of wood, which induces the formation of thermal‐stress‐induced microcracks and increases the ablation depth. Microcracks and increased ablation depth will reduce the electrical conductivity of the film produced by LIG. When the writing speed increases, heat induced microcracks will gradually disappear because of the weaker thermal accumulation in OHR region and thus the optimized electrical conductivity can be obtained (10 Ω sq^−1^ at 10 mm s^−1^ and a laser power of 0.8 W) in this region. With the writing speed increases, the thickness of graphene decreases (bottom half of Figure 12d) and the quality of laser induced graphene deteriorates, resulting in the increase of sheet resistance. The ablation depth and the thickness of laser induced graphene in each region is illustrated in Figure 12e. LIG can be accomplished using lasers with various wavelengths, including ultraviolet^[^ ^294^ ^]^ and visible 405 nm lasers.^[^ ^295^ ^]^ Due to the different light absorption efficiency of carbon precursors toward the wavelength of laser, the electric conductivity of LIG produced BCMs varies. For instance, leaf derived carbon materials produced by UV (346 nm) and green (520 nm) femtosecond lasers have different sheet resistances of 24.8 and 27.6 Ω sq^−1^, respectively.^[^ ^296^ ^]^

Morosawa et al. fabricated graphitic carbon by LIG derived from CNF films.^[^ ^297^ ^]^ The preparation process is illustrated in Figure 12f. The morphology of CNF films obtained from LIG is shown in Figure 12g. CNF‐derived carbon exhibited high conductivity up to 6.9 S cm^−1^, which can contribute to the formation of highly crystalline graphitic carbon, as demonstrated by the Raman spectra in Figure 12c. Another advantage of LIG is that designed patterns can be directly written on biomass precursors, which makes it possible to prepare BCMs with versatile functions. Zang et al. developed a direct‐write laser patterning process to prepare conductive molybdenum carbide‐graphene (MCG) composites directly on paper substrates.^[^ ^298^ ^]^ As‐prepared MCG has potential applications in 3D foldable paper electronics due to its strong mechanical stability and foldable property. Figure 12h shows an example of square‐shaped MCG electrodes fabricated on one paper that can be folded repeatedly. The LIG preparation process of MCG and the conversion process of paper substrates are shown in Figure 12i.

#### 4.2.4 Other Carbonization Methods

**Figure 13:**
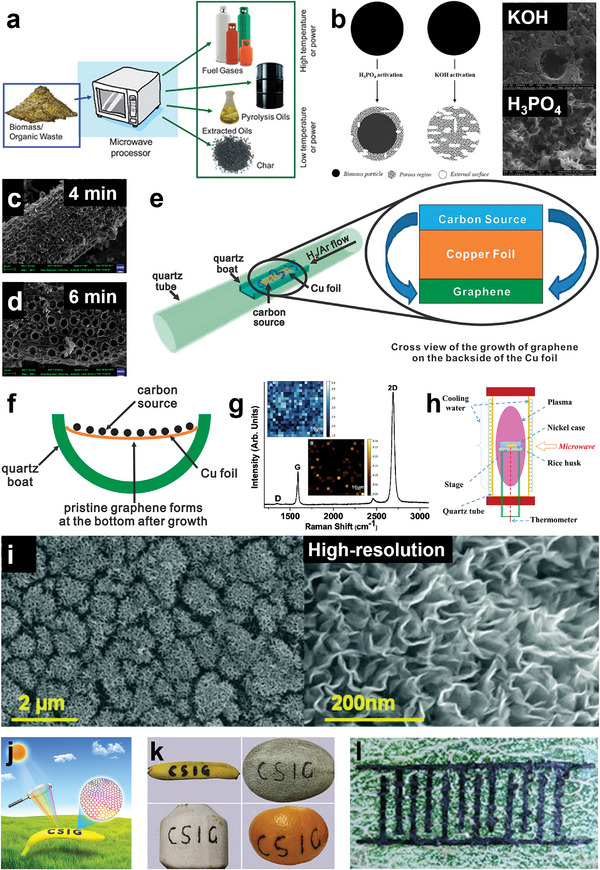
a) Illustration of MAP concept. Reproduced with permission.^[^ ^302^ ^]^ Copyright 2012, RSC. b) Pore development schematic diagrams and SEM photographs of H_3_PO_4_ or KOH activated CPH carbon. Reproduced with permission.^[^ ^305^ ^]^ Copyright 2019, ACS. SEM of oil palm male flowers derived nanoporous carbons by microwave‐assisted activation: c) 4 min and d) 6 min of irradiation time. Reproduced with permission.^[^ ^306^ ^]^ Copyright 2020, The authors, MDPI. e) Schematic diagram of the experimental apparatus for the growth of graphene from biomass. Reproduced with permission.^[^ ^308^ ^]^ Copyright 2011, ACS. f) Diagram of the graphene growth in the apparatus. Reproduced with permission.^[^ ^308^ ^]^ Copyright 2011, ACS. g) Raman spectrum and Raman spectral mapping (scanning was performed at every 5 µm over an area of 100 µm × 100 µm.) of 2D/G, D/G ratio in dog feces derived graphene. Reproduced with permission.^[^ ^308^ ^]^ Copyright 2011, ACS. h) Diagram of the setup for MPI method. Reproduced with permission.^[^ ^309^ ^]^ Copyright 2015, Elsevier. i) SEM and high‐resolution SEM images of honeycomb derived graphene. Reproduced with permission.^[^ ^310^ ^]^ Copyright 2015, RSC. j) Schematic diagram of concentrated solar induced graphene preparation. Reproduced with permission.^[^ ^312^ ^]^ Copyright 2022, The authors, RSC. k) Photograph of concentrated solar induced graphene patterned into a shape of the letters “CSIG” on banana peel, cantaloupe peel, coconut peel, and orange peel. Reproduced with permission.^[^ ^312^ ^]^ Copyright 2022, The authors, RSC. l) Image of concentrated solar induced graphene patterned into 12 interdigital electrodes with a line width of ≈2 mm on cantaloupe peel. Reproduced with permission.^[^ ^312^ ^]^ Copyright 2022, The authors, RSC.

Microwave‐assisted pyrolysis (MAP) is an emerging technology to convert biomass into BCMs in recent years. MAP uses electromagnetic waves (EMWs) with wavelengths of 1 mm to 1 m and frequencies ranging from 300 MHz and 300 GHz to directly deliver heat via molecular interactions.^[^ ^300^ ^]^ The concept of MAP is illustrated in **Figure** **13**a. MAP has several advantages, such as reduced processing times due to dielectric heating, which directly transfers electromagnetic energy to thermal energy.^[^ ^301^ ^]^ In addition, pretreatment, such as drying, can be reduced because water can be removed during MAP because of the polar nature of water.^[^ ^302^ ^]^ The processing parameters of MAP, such as irradiation time and energy density, are easy to control.^[^ ^303^ ^]^ However, MAP also has limits on the feedstock, and the addition of additional microwave absorbents is always necessary.^[^ ^304^ ^]^ MAP can be combined with chemical activation methods to fabricate porous BCMs. Villota et al. prepared waste cocoa pod husk‐derived porous carbon via microwave‐assisted activation by H_3_PO_4_ or KOH.^[^ ^305^ ^]^ H_3_PO_4_ is a better activation agent than KOH in all cases considered microwave‐assisted activation. According to the comparative study on the pore development of both activators, KOH is more active during the preparation process, as proven by the severe material loss and low structural integrity, as shown in the pore development schematics and SEM photographs of Figure 13b. The microwave irradiation power and time have a large influence on the pore characteristics of BCMs prepared by MAP combined activation according to the study by Kaewtrakulchai et al.^[^ ^306^ ^]^ They prepared oil palm male flower‐derived nanoporous carbons by MAP combined with KOH activation. When the irradiation time increased from 4 min to 6 min, the SSA of the carbon material increased from 911 to 991 m^2^ g^−1^. The morphology of the as‐fabricated carbons also varied vastly, and more pores can be observed on the 6 min irradiated sample (Figure 13c,d).

Recently, CVD growth of monolayer graphene from food, insects and waste has been reported by researchers.^[^ ^307^ ^]^ Ruan et al. developed a cost‐effective approach to grow graphene from low‐valued biomass waste on the backside of a Cu foil at 1050 °C under H_2_/Ar flow.^[^ ^308^ ^]^ The setup schematic of CVD growth is presented in Figure 13e. Carbon feedstock is placed atop a copper foil in a quartz boat placed in a tubular furnace. After CVD growth, graphene only forms on the backside of the Cu foil, as shown in Figure 13f. In addition, the graphene obtained from food, insects and wastes is of good quality. Figure 13g is the Raman spectrum of graphene from dog feces. The D band is trace in the spectrum while the G and 2D bands are located at 1585.5–1591.4 and 2682.6–2693.9 cm^−1^, respectively. The full width at half maxima values of *G* and 2D bands are 14.1–16.3 and 32.0–35.1 cm^−1^, respectively. The *I*_2D_/*I*_G_ value is calculated to be ≈4. Inserted in the Raman spectrum are Raman spectral mapping (mapping area 100 µm × 100 µm) of *I*_2D_/*I*_G_ and *I*_D_/*I*_G_. Over 95% of the scanning area in the dog feces case has the signature of *I*_2D_/*I*_G_ > 1.8 and *I*_D_/*I*_G_ < 0.1. The result of Raman spectroscopy demonstrates the formation of monolayer graphene.

Plasma has been employed to convert biomass into BCMs. The carbon products obtained from this method can be both graphene and CNTs.^[^ ^243^ ^]^ Wang et al. developed a microwave plasma irradiation (MPI) method to prepare nanocarbons from rice husk (RH), and the preparation apparatus is presented in Figure 13h.^[^ ^309^ ^]^ By the MPI method, the products include graphene, CNTs, and grapheneated CNTs (g‐CNTs). Their study also indicates that the pyrolysis of RHs and the formation of graphitic carbons strongly depend on the temperature and pressure during MPI. Seo et al. fabricated graphene through Ar/H_2_ plasma from a honeycomb for supercapacitors and biosensors.^[^ ^310^ ^]^ It is worth noting that the unique features of plasma, such as the electronic field in the plasma sheath, can guide the vertical growth of graphene (Figure 13i). Shah et al. pointed out that a long exposure time can lead to the formation of monolayer graphene, which is exfoliated from multilayer graphene formed under a short exposure time when using plasma to synthesize graphene from mango peel.^[^ ^311^ ^]^ In addition, when adopting this method, precautions are necessary due to the etching effect of plasma.

In a recent study, Hu et al. proposed a facile, green and fast method to prepare BCMs via concentrated solar radiation.^[^ ^312^ ^]^ The basic principle of this method is photothermal conversion. The sunlight can be concentrated to form a focused light spot via a biconvex. The heating temperature is over 1000 °C, which is able to directly convert biomass to graphene in a short time of 2–3 s. This process is illustrated in Figure 13j. In addition, this method can be applied to various biomass precursors, such as banana peel, cantaloupe peel, coconut peel, and orange peel, and the shape of graphene is also designable, as shown in Figure 13k,l.

### 4.3 Applications of Machine Learning in the Precise Control of Biomass‐Derived Carbon Materials Preparation

As mentioned above, BCMs can be synthesized from hundreds of biomass precursors via various preparation methods and their morphology, structure and porosity of the final products are influenced by tens of synthesis parameters such as temperature, time, heating rate, and additives. Therefore, it is essential to statistically analyze the relationship between the process conditions and the properties of the final products and build predictive models to achieve the controlled synthesis of BCMs. This requires continuous synthesis and characterization, which is rather time‐ and lab‐ consuming.^[^ ^313^ ^]^ With such large amounts of parameters to process, experimental trial and error approach is no longer practical.

**Figure 14:**
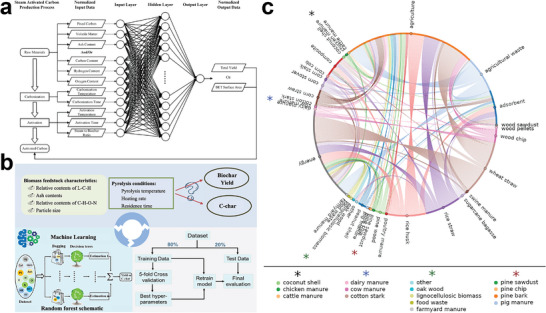
a) The structure of the multi‐layer feedforward ANN models to predict the total yield and the SSA of biochar. Reproduced with permission.^[^ ^319^ ^]^ Copyright 2019, The authors, Society of Chemical Industry and John Wiley & Son Ltd. b) The structure of the RF models to predict the yield and the C content of biochar. Reproduced with permission.^[^ ^323^ ^]^ Copyright 2019, Elsevier. c) Chord plot of the correlation between biomass precursors and their applications labeled as “adsorbent,” “agriculture,” “composite,” “energy,” and “other.” Reproduced with permission.^[^ ^324^ ^]^ Copyright 2022, ACS.

Thus, the machine learning (ML) method provides new opportunities for the controllable synthesis of BCMs because of its ability to learn behaviors and trends from available data without knowing the underlying physical mechanisms.^[^ ^314^ ^]^ In brief, ML is a data‐driven method that can build up relationships between variables using training data and appropriate algorithms to predict the structure or property of a material.^[^ ^315^, ^316^ ^]^ In recent years, algorithmic models based on ML such as artificial neural network (ANN) and random forest (RF), have been established to predict properties and performances of the BCMs according to the feedstock characteristics and processing conditions. ANN, one of the so called “black box” models, is able to reveal complicated relationships between multiple inputs and outputs without knowing the mathematical description of the phenomena.^[^ ^317^ ^]^ Djandja et al. developed a prediction model for the N content in carbonaceous materials prepared from sewage sludge through HTC.^[^ ^318^ ^]^ 138 data points from 26 published papers with regard to the elemental composition of the feedstock and HTC processing conditions were employed. The results show that the reaction temperature and the nitrogen, carbon, volatiles and fixed carbon contents of the raw materials are the most significant variables, among which the N content contributes the most. Liao et al. reported a multi‐layer feedforward ANN models to predict the yield and SSA of biomass derived activated carbon.^[^ ^319^ ^]^ The structure of this ANN model is shown in **Figure** **14**a. The trained model has good accuracy with mean square error (*R* ^2^) > 0.9 and the contribution analysis shows the large impact of activation conditions and feedstock properties on the yield and SSA of the final products. ANN can also be used to predict the performance of BCMs. For example, Yuan et al. applied ANN to systematically map the CO_2_ adsorption as a function of the textural and compositional properties of biomass waste‐derived porous carbons and adsorption parameters based on 527 data points including textural properties, the presence of multifarious functional groups, and the different temperatures and pressures to which they are subjected during CO_2_ adsorption.^[^ ^320^ ^]^ The optimized model exhibits good predictive performance with *R* ^2^ of 0.84 on the test data. Moreover, adsorption parameters, textural properties, and compositional properties are revealed as significant factors for BWDPC‐based CO_2_ adsorption ability in the order of precedence.

Random forest (RF) is another machine learning method that has been adopted in new material design and discovery. RF is a data mining method that can process the non‐linear relationship between variables based on classification and regression tree.^[^ ^321^ ^]^ RF has several statistical merits including the minimal risk of overfitting, fewer parameters which need to be specified and the capability for contribution analysis of each feature.^[^ ^322^ ^]^ Zhu et al. applied RF to predict the yield and C content of biochar prepared from lignocellulosic biomass and to determine the relative importance of the influencing factors including biomass feedstock characteristics and pyrolysis conditions.^[^ ^323^ ^]^ The structure of this RF model is illustrated in Figure 14b. The accuracies for predicting char yield and C content are up to 0.8548 and 0.8480 respectively using *R* ^2^ as criteria. Compared with biomass characteristics, pyrolysis conditions show higher contribution for yield (65%) and C content (53%).

ML method can help with the experimental design of the preparation of BCMs. For example, Paula et al. used an automatic reading‐interpreting‐extracting computational routines (the a.RIX engine) to build up a plat form for rational synthesis design of BCMs.^[^ ^324^ ^]^ 10 975 published from the years of 2000 to 2020 were processed using the a.RIX engine and more than a hundred precursors, synthesis conditions and parameters related to the BCMs’ properties were extracted from the papers. It is found that a correlation exists between biomass precursors and the application of biomass categorized as “adsorbent,” “agriculture,” “composite,” “energy,” and “other,” as shown in Figure 14c. Peanut shell‐derived BCMs have higher SSA than other precursor derived BCMs according to the statistic result. The results also show general trends in the correlations between BCMs synthesis conditions and their properties: First, the H/C and O/C ratios decrease with the increase of the carbonization temperature. Second, a high aromatic degree (low H/C ratio) and a low oxidation level (low O/C ratio) are beneficial for the synthesis of BCMs with high SSA.

## 5 Applications of Biomass‐Derived Carbon Materials

### 5.1 Applications in Energy‐Related Fields

#### 5.1.1 Biomass‐Derived Carbon Materials as Electrocatalysts

**Figure 15:**
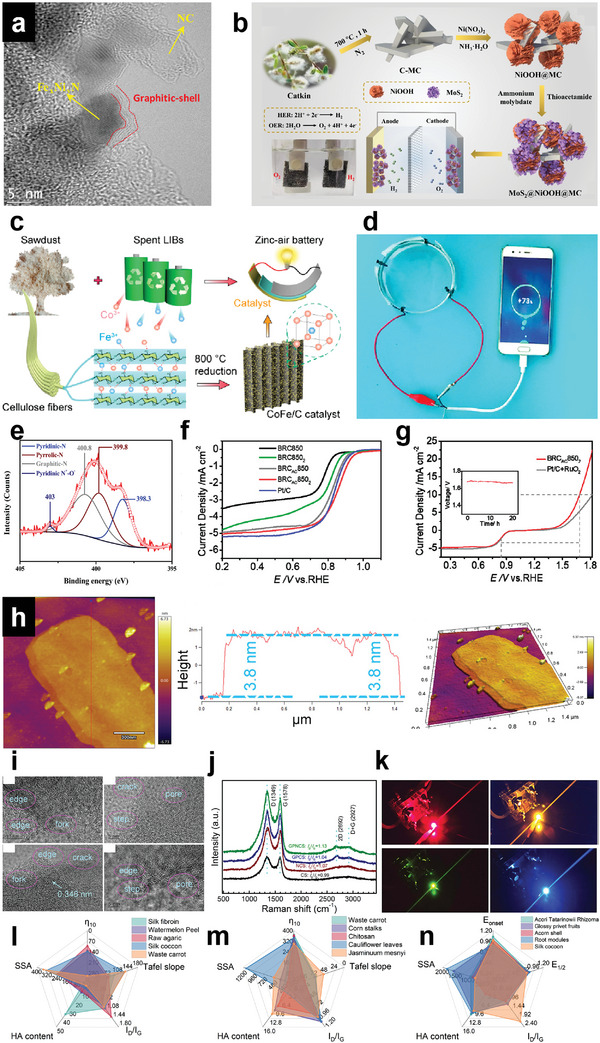
a) TEM image of Fe*_x_* Ni*_y_* N@C/NC. Reproduced with permission.^[^ ^326^ ^]^ Copyright 2020, WILEY‐VCH. b) Schematic of the preparation process of MoS_2_@NiOOH@C‐MC. Reproduced with permission.^[^ ^327^ ^]^ Copyright 2021, Elsevier. c) Illustration of the CoFe/C catalyst for ZAB. Reproduced with permission.^[^ ^328^ ^]^ Copyright 2022, ACS. d) A mobile phone charged by CoFe/C assembled ZAB. Reproduced with permission.^[^ ^328^ ^]^ Copyright 2022, ACS. e) XPS N1 s spectra of activated carbon nanosheets derived from peanut shells. Reproduced with permission.^[^ ^335^ ^]^ Copyright 2019, Elsevier. f) ORR polarization curves of catalysts at 1600 rpm in O_2_‐saturated 0.1 m KOH. Reproduced with permission.^[^ ^336^ ^]^ Copyright 2019, ACS. g) OER polarization curves of catalysts at 1600 rpm in O_2_‐saturated 0.1 m KOH. Reproduced with permission.^[^ ^336^ ^]^ Copyright 2019, ACS. h) AFM images and corresponding height analysis profiles of GPNCS. Reproduced with permission.^[^ ^344^ ^]^ Copyright 2020, ACS. i) TEM images displaying various defects of GPNCS. Reproduced with permission.^[^ ^344^ ^]^ Copyright 2020, ACS. j) Raman spectra of CS, GPCS, NCS, and GPNCS. Reproduced with permission.^[^ ^344^ ^]^ Copyright 2020, ACS. k) Red, yellow, green, and blue LEDs powered by two‐series batteries based on GPNCS. Reproduced with permission.^[^ ^344^ ^]^ Copyright 2020, ACS. l) Radar chart of BCMs‐based HER catalysts.^[^ ^158^, ^345^, ^346^, ^347^, ^348^ ^]^ m) Radar chart of BCMs‐based OER catalysts.^[^ ^347^, ^349^, ^350^, ^351^, ^352^ ^]^ n) Radar chart of BCMs‐based ORR catalysts.^[^ ^196^, ^336^, ^344^, ^353^, ^354^ ^]^

Electrochemical reactions such as the HER, OER, and ORR, normally occur in the water electrolysers, fuel cells, or metal–air batteries. The use of electrocatalysts can significantly improve these reaction processes. However, catalysts with high activity are mostly noble metals, such as Pt, Ru, and their compounds, which are expensive and rare. Transition metals have comparative catalytic performance but are with lower price and abundant resources and thus are considered as promising substitutes for noble metal catalysts. Nevertheless, these transition metal‐based electrocatalysts have significant drawbacks, such as their susceptibility to aggregation which will lead to poor interactions between catalysts and electrolytes as well as reduced exposure of active sites.^[^ ^325^ ^]^ This can be alleviated by using carbon supports such as BCMs, which have high conductivity, porous structures, and chemical tolerance.^[^ ^195^ ^]^ Wu et al. fabricated graphitic‐shell encapsulated FeNi alloy/nitride nanocrystals on cellulose‐derived N‐doped carbon as electrocatalysts for zinc‐air batteries (ZABs).^[^ ^326^ ^]^ This carbon support structure provides a direct charge transfer pathway and thus enhances the catalytic performance toward OER and ORR. The obtained Fe*_x_* Ni*_y_* N@C/NC sample exhibited superb bifunctional catalytic activity with the lowest value of the difference between the ORR and OER metrics (Δ*E* = *E*_j_ = 10‐ *E*_1/2_ = 0.67 V, where *E*_j_ = 10 refers to the potential at a current density of 10 mA cm^−2^ and *E*_1/2_ refers to the half wave potential). In addition, the metallic alloy nanocrystal compounds are encapsulated in the graphitic shell (demonstrated by TEM images in **Figure** **15**a), which protects the metal core from corrosion by the electrolytes and thus endows the catalysts with high stability. Liu et al. prepared catkin‐derived mesoporous carbon as a support for molybdenum disulfide (MoS_2_) and nickel hydroxyl oxide hybrid (NiOOH) for the catalysis of HER and OER, as shown in Figure 15b.^[^ ^327^ ^]^ The as‐prepared catalysts exhibit high catalytic activity toward HER and OER with a low potential of −250 mV for HER and 1.51 V for OER at the current density of 10 mA cm^−2^, which can be attributed to the different functions of MoS_2_ and NiOOH. MoS_2_ has better catalytic performance toward the HER, while NiOOH is an efficient OER catalyst. In addition, the carbon matrix also provides fast electron transfer capability, which is beneficial for electrocatalysis. For better catalytic performance, uniform dispersion of metal particles on the carbon matrix is needed. Jiao et al. reported BCM‐supported Co‐Fe intermetallic catalysts for the ORR.^[^ ^328^ ^]^ Based on the concepts of recycling and sustainability, Co and Fe are extracted from spent lithium‐ion batteries, and the carbon matrix is derived from saw dust. Due to the electrostatic attraction between Co^3+^ and Fe^3+^ cations and hydroxyl groups in saw dust, Co and Fe nanoparticles are uniformly dispersed in the carbon support with a unique alloy structure in which Fe atoms are isolated into single sites by Co atoms, as shown in Figure 15c. This structure endows the CoFe/C catalysts with a Pt‐like dissociative mechanism, leading to superior ORR catalytic performance with a large half‐wave potential of 0.85 V (0.83 V for Pt/C catalysts). When assembled in ZAB, the CoFe/C catalyst exhibits a remarkable power density of 199.2 mW cm^−2^, which is able to charge a mobile phone (depicted in Figure 15d). It is also impressive that this catalyst is of extremely low cost, with 0.71 $ g^−1^ compared with 85.45 $ g^−1^ for commercial Pt/C and 28.21 $ g^−1^ for RuO_2_.

Due to the molecular structure of biomass precursors, BCMs are usually doped with heteroatoms, such as N, S, O, and P. Heteroatom‐doped carbon materials, such as N‐doped carbon, have been demonstrated to have good catalytic performance even comparable to noble metal catalysts by Dai et al. in 2009^[^ ^329^ ^]^, which stimulated the research interests of exploiting metal‐free heteroatom‐doped carbon catalysts. With heteroatom doping, the charge density and electronic distribution of the resulting BCMs can be adjusted, leading to the enhanced electrocatalytic ability.^[^ ^330^, ^331^ ^]^ Both carbon atoms adjacent to the dopant and the dopant atom itself can act as active sites in heteroatom doped carbon materials.^[^ ^332^ ^]^ In addition, by co‐doping different heteroatoms, heteroatom doped carbon materials can have multiple catalytic functionalities due to the co‐existence of various active sites.^[^ ^333^ ^]^ Nevertheless, the application of heteroatom‐doped carbon materials derived from fossil‐based feedstocks, such as CNTs and graphene, is hindered due to the cost concerns.^[^ ^334^ ^]^ Therefore, BCMs can be sustainable and low‐cost candidates for developing metal‐free carbon catalysts. Our group has prepared silk fibroin‐derived porous carbon nanofibers by electrospinning and chemical activation as N‐doped metal‐free electrocatalysts for the HER in both acidic and alkaline solutions.^[^ ^158^ ^]^ Due to the high N content, hierarchical porous structure produced by chemical activation and large through pores between carbon fibers, the 4%‐SPCNF sample exhibits the best catalytic activity among its rivals, with overpotentials of 310.86 ± 12.93 mV and 401.3 ± 7.92 mV at a current density of 10 mA cm^−2^ in acidic and alkaline electrolytes, respectively. Nitrogen self‐doped activated carbon nanosheets derived from peanut shells were synthesized by Saravanan et al. for catalysis toward the HER.^[^ ^335^ ^]^ The doped N content is 6.66% and exists in the carbon network in various types, including pyridinic‐N and pyrrolic N, as demonstrated by XPS in Figure 15e. This activated BCM also has enhanced HER performance with a low onset potential of 80 mV. Li et al. developed a top‐down strategy to prepare 3D metal‐free porous carbon derived from Acori Tatarinowii Rhizoma, a traditional Chinese medicine, as metal‐free bifunctional electrocatalysts for OER and ORR in ZABs.^[^ ^336^ ^]^ The catalyst BRC_AC_850_2_ has excellent electrocatalytic performance toward both the ORR with an E_1/2_ of 0.85 V (Figure 15f) and OER with *E*_j = 10_ of 1.68 V versus RHE (Figure 15g), which even outperforms the activity of commercial Pt/C and RuO_2_. The bifunctional catalytic activity can be attributed to the 3D hierarchical structures and rich active sites of the catalyst, including N functional groups, oxygen vacancies, and carbon defects. It is noteworthy that oxygen vacancies have been demonstrated to play a positive role in enhancing catalytic performance of the electrocatalysts by the following aspects: 1) Oxygen vacancies can change the electron distribution on the surface of the materials and thus decreases the adsorption energy of reaction intermediates, H_2_O molecules in OER for example. This effect will in turn leads to the acceleration of the reaction.^[^ ^337^ ^]^ 2) The introduction of oxygen vacancies can enhance the electrical conductivity of the materials and promote charge transfer process.^[^ ^338^ ^]^ 3) Oxygen vacancies are representative of electron donors. Their formation can modify the electronic structure of the catalyst and change the rate‐determining step of the electrochemical reaction.^[^ ^339^ ^]^

Apart from heteroatom doping, intrinsic edge and topological defects have major impact on the catalytic activity of BCMs.^[^ ^340^ ^]^ Defects in carbon can optimize the surface electronic properties and therefore improve the kinetic catalytic activity of oxygen‐related reactions.^[^ ^341^ ^]^ For example, the charge density of the carbon atoms locating at the edge sites can be elevated, carrying high positive charge. These carbon atoms with positive charge can be potential active sites for electrocatalysis.^[^ ^342^ ^]^ Electron transfer can be induced by the defects on carbon materials, resulting in the enhanced electrical conductivity of the BCMs.^[^ ^343^ ^]^ Liu et al. fabricated graphene‐like and defect‐rich carbon sheets from glossy privet fruits as electrocatalysts for rechargeable ZABs (RZABs).^[^ ^344^ ^]^The nanosheets have an ultrathin structure with a monolayer thickness of ≈2 nm, as determined by atomic force microscopy, as shown in Figure 15h. The defect‐rich property of the catalysts can be demonstrated by TEM images (Figure 15i) with multiple defective structures, such as edges, pores, forks, and cracks, and Raman spectra (Figure 15j) with high *I*_D_/*I*_G_ values. The synergetic effect of both N doping and topological defects endows graphene‐like and defect‐rich carbon sheets with N doping (GPNCS) with a superb catalytic performance of an ORR onset potential of 0.92 V and E_1/2_ of 0.81 V. It is noteworthy that two RZABs based on GPNCS can power a light‐emitting diode (LED) that starts at a minimum potential of 2.2 V, as shown in Figure 15k.

Herein, we have summarized the performance and structural properties of BCM‐based electrocatalysts for the HER, OER, and ORR in recent literature using radar charts. Figure 15l, m and n are radar charts of BCMs‐based HER, OER, and ORR catalysts, respectively. The benchmark parameters chosen in radar charts for catalytic performance toward HER and OER are *η*_10,_ which refers to the overpotential at a current density of 10 mA cm^−2,^ and the Tafel slope, which is related to the reaction kinetics. For ORR, the chosen parameters for catalytic activity are *E*_onset_ and *E*_1/2_. The structural properties chosen in radar charts are the SSA related to the porous structure, heteroatom content (HA content) for the HA doping effect and I_D_/I_G_ standing for defects. It can be concluded that the performance of the catalysts is not solely related to one of the structural properties but has complex interactions with defects, porous structures, and heteroatom doping.

#### 5.1.2 Biomass‐Derived Carbon Materials in Electrochemical Energy Storage Devices

Energy storage devices (EESDs), including supercapacitors and rechargeable batteries, have attracted wide attention of researchers worldwide due to their superb ability to store and release electric power efficiently and reversibly via electrochemical process.^[^ ^355^, ^356^ ^]^ EESDs have been widely used in important applications such as portable electronic devices, electric vehicles (EVs), and aviation aircrafts. A series of materials for EESDs, including metals, conductive polymers, and carbon materials, have been investigated during the past decades.^[^ ^357^ ^]^ For the application in wearable electronic devices and the rapid development of EVs, materials for EESDs not only should satisfy the energy density and power density demands of EESDs but also should meet the specific requirement such as flexibility, light‐weight, stretchability, fast‐charging, and small size, for certain applications.^[^ ^358^ ^]^ Carbon‐based materials, including BCMs, show great potential in the development of advanced EESDs due to the following advantages: 1) Compared with other materials, the properties of carbon materials, such as SSA, pore structure, electric conductivity, and electrochemical/chemical stability, can be easily tailored by the manipulation of their dimensionality.^[^ ^359^ ^]^ 2) Carbon materials usually have great mechanical strength, superb flexibility, light weight, and superior processability and can be assembled in flexible or stretchable EESDs.^[^ ^360^, ^361^ ^]^ 3) Carbon materials can either be effectively composited with other functional materials, for instance elastic polymers, to fabricate flexible electrodes or act as support materials for active materials (e.g., metal nanoparticles) to boost the electrochemical performance.^[^ ^359^, ^362^ ^]^ In this section, the recent progress of BCMs in the application of EESDs and strategies to boost their performance are presented.

##### Biomass‐Derived Carbon Materials in Supercapacitors

**Figure 16:**
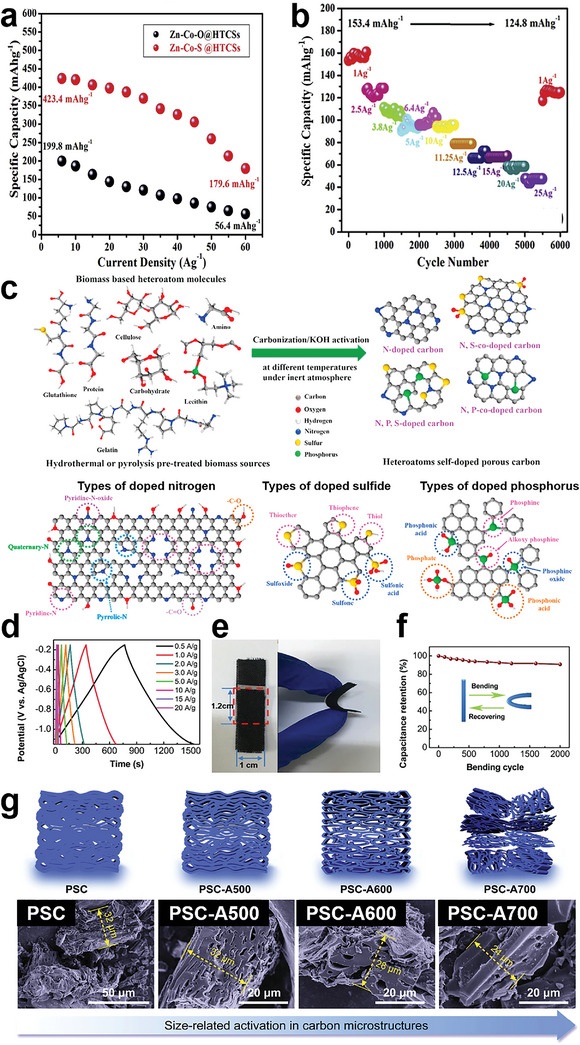
a) Dependence of the gravimetric capacity of Zn‐Co‐S@HTCSs‐NF on the applied current density. Reproduced with permission.^[^ ^366^ ^]^ Copyright 2019, Elsevier. b) Capacitance variation of the fabricated ASCs as a function of cycle number measured at different current densities. Reproduced with permission.^[^ ^366^ ^]^ Copyright 2019, Elsevier. c) Schematic illustration of heteroatom self‐doped porous carbon derived from biomass sources. Reproduced with permission.^[^ ^363^ ^]^ Copyright 2020, Elsevier. d) GCD curves of PAN/Konjac‐800‐1 at various current densities of 0.5–20 A g^−1^. Reproduced with permission.^[^ ^369^ ^]^ Copyright 2020, ACS. e) Photo of a flexible solid‐state supercapacitor. Reproduced with permission.^[^ ^369^ ^]^ Copyright 2020, ACS. f) Capacitance retention after 2000 cycles of bending. Reproduced with permission.^[^ ^369^ ^]^ Copyright 2020, ACS. g) Morphology evolution of pencil shaving derived carbon through different activation temperature. Reproduced with permission.^[^ ^373^ ^]^ Copyright 2020, Elsevier.

Supercapacitors have attracted massive attention due to their long cycling life, rapid charge/discharge rate, high power density, environmental benignancy, and low maintenance cost.^[^ ^363^ ^]^ Carbon materials are desirable for the electrode materials of electrochemical double‐layer capacitors (EDLCs), which are one of the major types of supercapacitors working on the mechanism of nonfaradaic electrostatic charge storage at the electrode/electrolyte interface.^[^ ^364^ ^]^ For high‐performance supercapacitors, it is necessary to design the graphitization degree, SSA, and surface properties of electrode materials. As mentioned above, it is facile to prepare porous and heteroatom‐doped carbon materials from biomass precursors. Therefore, BCMs are sustainable, low‐cost, and nontoxic materials for supercapacitors. To regulate the pore structure of BCMs for enhanced supercapacitor performance, Xu et al. proposed a dual‐porogen synthesis strategy by pyrolysing C_10_H_14_N_2_Na_2_O_8_/KOH (dual‐porogen) and walnut peel together with HCl solution etching.^[^ ^365^ ^]^ The pore size distribution is opened up to 0.59–2.53 nm from 0.55–1.76 nm due to such synthesis strategy. The carbon materials with optimized fabrication conditions exhibit a high capacitance of 557.9 F g^−1^ (at 1 A g^−1^) and 291.0 F g^−1^ (at 30 A g^−1^) and power density up to 5679.62 W kg^−1^ and an energy density of 12.44 W h kg^−1^. Although EDLCs based on BCMs have high power density and outstanding cycle stability, their specific capacitance and energy density are lower than those of pseudocapacitors, which works on the mechanism of faradaic charge storage by redox reactions.^[^ ^365^ ^]^ One solution is to combine BCMs with pseudocapacitor materials such as metal oxides and sulfides. Hekmat et al. prepared zinc‐cobalt sulfide (Zn‐Co‐S) nanomaterials loaded on biomass‐derived hydrothermal carbon spheres on Ni foam (Zn‐Co‐S@HTCSs‐NF) as the positive electrode of asymmetric supercapacitors (ASCs) where Ni foam acts as the current collector.^[^ ^366^ ^]^ The SSA of Zn‐Co‐S@HTCSs‐NF is 106.8 m^2^ g^−1,^ and the average pore diameter is 20.6 nm. Additionally, due to the loading of Zn‐Co‐S nanomaterials, the specific capacity of the positive electrode is as high as 423.4 mAh g^−1^ (at 1 A g^−1^), and when the current density increases from 6 A g^−1^ to 40 A g^−1,^ the specific capacity retains 82% (**Figure** **16**a). The fabricated ASCs present a high specific capacity of 149 mAh g^−1^ (at 1 A g^−1^) and a superb high energy density (≈85 Wh.kg^−1^) at a reasonable power density of 460 W kg^−1^. Moreover, the cycling ability of the fabricated ASCs is promising, with 81.4% retention over 6000 charge–discharge cycles, as shown in Figure 16b.

Another solution to increase the intrinsic energy density and specific capacity of BCM‐based EDLCs is to dope heteroatoms into the carbon network. The introduced heteroatoms can influence the electronic, chemical, and mechanical properties of sp^2^ hybridized carbon due to the local curvature induced by doping, which increases the local reactivity. Therefore, the conductivity and surface wettability of carbon are optimized, and pseudocapacitance will also be induced.^[^ ^367^ ^]^ Heteroatom‐doped BCMs can be synthesized by direct pyrolysis or pretreatment of biomass sources, and the types of doped heteroatoms are summarized by Gopalakrishnan et al., as shown in Figure 16c.^[^ ^363^ ^]^ Li et al. synthesized a self‐doped carbon foam with 3D hierarchical pore structure from corn stalk via hydrothermal and pyrolysis.^[^ ^368^ ^]^ Due to the large amount of N/O components in corn stalk precursor, the obtained carbon foam has a N/O co‐doped structure. Besides, the activation of KOH endows the carbon foam with well‐developed porous structure with a high SSA of 1272 m^2^ g^−1^ and a low packing density of 0.83 g cm^−3^. Contributed by large SSA, hierarchical porosity and rich structural defects induced by KOH activation, the optimal carbon foam exhibits gravimetric and volumetric capacitances up to 282 F g^−1^ and 234 F cm^−3^ at 0.5 A g^−1^ respectively, and a high‐rate capacitance retention of 72.7% at a large rate of 100 A g^−1^. Bai et al. prepared konjac/PAN‐based nitrogen‐doped porous carbon for supercapacitors.^[^ ^369^ ^]^ By KOH activation, the SSA of the PAN/Konjac (1:1)‐800 sample reaches 2125 m^2^ g^−1,^ and the doped nitrogen content is 1.54 atom%. The galvanostatic charge/discharge curves of PAN/Konjac (1:1)‐800 indicate that charge storage and delivery are highly reversible, as depicted in Figure 16d. In addition, due to the large SSA and heteroatom doping, PAN/Konjac‐800‐1 materials exhibit a high capacitance of 390 F g^−1^, reasonable capacitance retention (from 0.5 to 20 A g^−1^) of 70% and good cycling life with 95.5% retention upon 10 000 charge/discharge cycles at 5 A g^−1^. It is noteworthy that the fabricated materials are flexible, as shown in Figure 16e. The device has great mechanical strength and flexibility with 91% capacitance retention after bending‐releasing for 2000 cycles (Figure 16f).

The recent years have witnessed the thriving of zinc ion hybrid supercapacitors (ZHSCs) due to the remarkable advantages of ZHSCs, such as high reliability and safety, low cost, noncorrosive, nontoxic nature.^[^ ^370^ ^]^ BCMs have been proved as promising positive electrode materials for the fabrication of ZHSCs.^[^ ^371^, ^372^ ^]^ Li et al. constructed a flexible and anti‐freezing quasi‐solid‐state ZHSC with cheap zinc foil as negative electrode and porous carbon derived from pencil shavings as positive electrode.^[^ ^373^ ^]^ The porous structure is obtained by KOH activation and can be optimized by tuning the carbonization temperature, as shown in Figure 16g. Owing to the large accessible surface area and numerous active sites for ions adsorption provided by the well‐defined structure of pencil shaving‐derived carbon electrode, the as‐built ZHSC exhibits a specific capacitance of 413.3 F g^−1^ at 0.2 A g^−1^ with a mass loading of 2.0 mg cm^−2^. The reported ZHSC also has superb durability with 92.2% retention after 10 000 cycles at a current density of 10 A g^−1^.

##### Biomass‐Derived Carbon Materials in Batteries

Metal‐ion secondary batteries (MSBs), such as lithium‐ion batteries and sodium‐ion batteries are considered promising and green energy storage devices. Numerous studies have been conducted to pursue batteries with high energy density and better safety. At the same time, researchers have also attempted to enhance the sustainability of batteries by replacing nonsustainable components with sustainable ones.^[^ ^374^ ^]^ Therefore, BCMs with designable structures and abundant resources show great potential for application in MSBs. The typical components of MSBs usually include anodes, cathodes, separators, electrolytes, binders, and additives. Herein, applications of BCMs in batteries as different components are introduced.

##### Biomass‐Derived Carbon Materials in Batteries—Biomass‐Derived Carbon Materials as Cathodes

**Figure 17:**
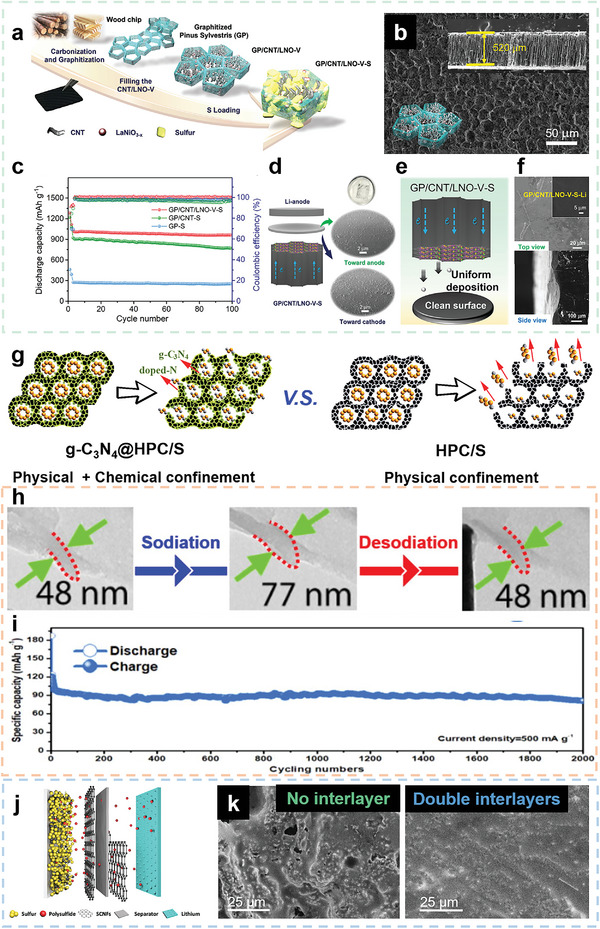
a) Preparation process of GP/CNT/LNO‐V‐S cathode. b) SEM morphology of GP/CNT/LNO‐V‐S. c) Cycle performance at 0.2 C with a sulfur loading of 4.4 mg cm^−2^. d) Digital photos of 50 cycled separators of cells with GP/CNT/LNO‐V‐S cathode. e) Schematics of Li metal deposition in cells with GP/CNT/LNO‐V‐S. f) Morphology of 50 cycled Li‐anode in the cell with GP/CNT/LNO‐V‐S from top view and side view. (a)–(f) Reproduced with permission.^[^ ^377^ ^]^ g) Schematics of the dual physical/chemical confinement of polysulfide species by g‐C_3_N_4_@HPC. Reproduced with permission.^[^ ^379^ ^]^ Copyright 2020, Elsevier. h) In situ TEM characterizations of sugarcane‐derived carbon. Reproduced with permission.^[^ ^381^ ^]^ Copyright 2022, Elsevier. i) Long‐term cycling performance of sugarcane‐derived carbon anode. Reproduced with permission.^[^ ^381^ ^]^ Copyright 2022, Elsevier. j) Schematic model of cells with double CNSF interlayers. Reproduced with permission.^[^ ^384^ ^]^ Copyright 2019, Elsevier. k) SEM images of Li surface with or without CNSF interlayers. Reproduced with permission.^[^ ^384^ ^]^ Copyright 2019, Elsevier.

Cathodes of MSBs are usually made of metal oxides. Taking lithium‐ion batteries as an example, widely used cathode materials include LiMnO_2_ and LiFePO_4_. These cathode materials exhibit stable capacity but have limitations for further improvement due to their low theoretical capacity.^[^ ^375^ ^]^ It is demonstrated that hard carbon (HC) materials with porous or 3D structures as the scaffold of cathodes can address this issue. Such a conductive scaffold not only provides an interconnected porous network that favors the absorption of electrolyte but also reduces the diffusion path of the lithium ions, thus improving the volumetric energy density and rate capability.^[^ ^376^ ^]^ Zou et al. used graphitized *Pinus sylvestris* (GP) as the scaffold for cathodes of LSBs.^[^ ^377^ ^]^ CNTs and LaNiO_3_‐*x* (LNO‐V) nanoparticles are filled in GP by vacuum filtration to accommodate sulfur without blocking the transport of Li ions. Furthermore, sulfur was permeated into GP/CNT/LNO‐V host materials to fabricate GP/CNT/LNO‐V‐S, as shown in **Figure** **17**a. The morphology of the composites is shown in Figure 17b. Compared with other cathodes prepared in this work, the GP/CNT/LNO‐V‐S cathode exhibits the highest discharge capacity of 1282 mAh g^−1^ and 5.64 mAh cm^−2^ at 0.05 C with a sulfur loading of 4.4 mg cm^−2,^ as shown in Figure 17c. The GP/CNT/LNO‐V‐S cathode also exhibits a superb long cycling performance with the highest capacity of 714 mAh g^−1^ (S loading: 4.4 mg cm^−2^, E/S: 6 µL mg^−1^) and the most stable capacity retention rate of 99% after 500 cycles. This result can be attributed to the physical confinement and highly electron/ion conductive structure of GP, which effectively suppresses the diffusion of intermediate polysulfides (LiPSs) and enhances the utilization of active materials. In addition, the confinement of GP can also hinder the shuttle effect of LiPSs, as presented in Figure 17d. The deposition of LiPSs on the anode can cause the formation of Li dendrites, and it is found that the GP scaffold is able to improve lithium deposition. The mechanism of Li metal deposition in cells with GP/CNT/LNO‐V‐S and the morphology of the Li anode are presented in Figure 17e,f, respectively. Apart from physical confinement, chemical confinement using highly polarized species (including metal oxides, nitrides, sulfides) possess a strong affinity to lithium polysulfides, which can anchor the polysulfides on the carbon matrix.^[^ ^378^ ^]^ Hong et al. proposed a method to produce graphitic carbon nitride@hierarchical porous wheat flour‐derived carbon (g‐C3N4@HPC) composites on a hundred‐gram scale as sulfur host materials for the cathodes of Li–S batteries.^[^ ^379^ ^]^ With both the physical confinement of the porous structure and chemical confinement of graphitic nitrides, g‐C3N4@HPC/S cathodes have strong chemical interactions to anchor LiPSs and thus reduce the shuttle effect of Li–S batteries, as shown in Figure 17g. The cycling stability of the g‐C3N4@HPC/S cathodes is excellent, with a very slight capacity decay of 0.024% per cycle for 250 cycles.

##### Biomass‐Derived Carbon Materials in Batteries—Biomass‐Derived Carbon Materials as Anodes

HC materials are promising anode materials for MSBs because of their superior physical and chemical stabilities, high reversible capacity, and great nontoxicity.^[^ ^380^ ^]^ Among various HC precursors, biomass is desirable due to its low cost and sustainability. Kim et al. fabricated porous carbon derived from low‐cost sugarcane biomass as anode materials for sodium‐ion batteries (NIBs).^[^ ^381^ ^]^ The optimized sample exhibits a high initial reversible capacity of 229 mAh g^−1^ as well as a reversible capacity of 189 mAh g^−1^ at 100 mA g^−1^ after 50 cycles. In situ TEM characterization indicates that the volumetric changes of the anode material are small during the sodiation–desodiation process (Figure 17h), suggesting the stable structure of the sugarcane‐derived carbon anode. Such structure endows the material with ultrastable capacity with almost no attenuation after 2000 cycles (Figure 17i). Zhao et al. synthesized 3D scaffolding S‐doped carbon nanosheets from plant biomass for Na‐ion batteries.^[^ ^382^ ^]^ The codoping of S‐O‐N, enlarged layer space and 3D porous structure not only offer abundant reaction sites and improve the conductivity but also lead to enhanced wettability, additional Faradaic reactions, convenient path for Na^+^ diffusion and robust host for Na^+^ insertion/extraction. The reversible capacity of the optimized sample reaches 605 mA h g^−1^ at 0.05 A g^−1,^ and the initial coulombic efficiency is as high as 58%. In addition, the anode materials exhibit superb long‐term stability of 211 mA h g^−1^ at a current density of 5 A g^−1^ after 2000 cycles, and the capacity retention is 94%.

##### Biomass‐Derived Carbon Materials in Batteries—Biomass‐Derived Carbon Materials in Separators

The functions of separators in MSBs are to isolate cathodes and anodes from short circuits and to enhance the ion transport efficiency as well as to resist the formation of dinitride or the shuttle effect.^[^ ^383^ ^]^ The commercialized separators are polypropylene (PP) and polyethylene (PE) polymers, which are light, thin and porous. However, these separators still have disadvantages, such as poor mechanical properties and thermal stabilities. BCMs have been fabricated to improve the performance of battery separators either as the matrix materials or inter protective layers. Wu et al. fabricated a carbon interlayer of separators for LSBs from regenerated silk fiber (CNSF).^[^ ^384^ ^]^ With both the cathode and anode interlayers, the long‐term cycling performance is enhanced with a reversible capacity of 799 mA h g^−1^ after 200 cycles at 0.2 C and an average capacity loss of 0.018% per cycle. There are multiple functions of the interlayer materials. First, the conductive property of CNSF offers a fast electron pathway through the insulating S/Li_2_S. Second, the porous structure of CNSF can physically block dissolved polysulfide. In addition, the anode interlayer can suppress the formation of Li dendrites and the corrosion of Li. The model of the protection mechanisms of the CNSF interlayer is shown in Figure 17j. SEM images clearly demonstrated the protection of CNSF interlayers for the suppression of the formation of Li dendrites and Li corrosion. Compared with cells without an interlayer, only small damage can be observed on the Li surface of cells with double interlayers, as presented in Figure 17k.

### 5.2 Applications in Electronics

Recent decades have witnessed the emergence and flourishment of research on soft electronics, which are tiny, lightweight, flexible, and stretchable.^[^ ^385^ ^]^ Soft electronics can be applied to healthcare monitors, human‐machine interactions, and intelligent robots.^[^ ^386^ ^]^ With high thermal and chemical stability, great conductivity and fast electron mobility, carbon materials are considered a suitable matrix for soft electronics. To pursue a sustainable way of producing carbon materials for soft electronics, biomass is widely selected as the carbon precursor. BCMs possess various advantages apart from sustainability, such as multiplex design of structure and morphology, facile functionalization ability, and tunable surface and electron structure.^[^ ^387^ ^]^ At present, BCMs have been used in different types of soft electronics, including sensors and e‐textiles.

#### 5.2.1 Applications in Sensors

##### Biomass‐Derived Carbon Materials as Piezoresistive Sensors

**Figure 18:**
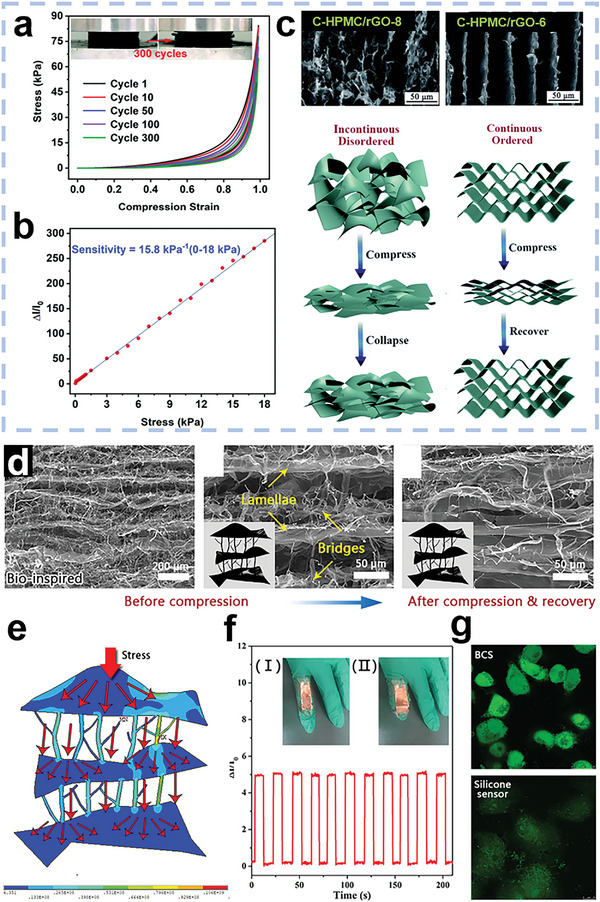
a) Stress–strain curves of C‐HPMC/rGO‐6 at 99% strain for 300 cycles. Reproduced with permission.^[^ ^389^ ^]^ Copyright 2020, RSC. b) Linear sensitivity of C‐HPMC/rGO‐6 in a wide pressure range of 0–18 kPa. Reproduced with permission.^[^ ^389^ ^]^ Copyright 2020, RSC. c) Morphology and elastic mechanisms of C‐HPMC/rGO‐8 and C‐HPMC/rGO‐6. Reproduced with permission.^[^ ^389^ ^]^ Copyright 2020, RSC. d) SEM images of BCS before and after 100 cycles of compression. Reproduced with permission.^[^ ^390^ ^]^ Copyright 2020, The authors, WILEY‐VCH. e) Pressure spreading schematics over BCS. Reproduced under terms of the CC‐BY license.^[^ ^390^ ^]^ Copyright 2020, The authors, WILEY‐VCH. f) Current changes (Δ*I*/*I*_0_) under different progressive motions (I for straight and II for bending) of the piezoresistive sensor on a finger. Reproduced with permission.^[^ ^390^ ^]^ Copyright 2020, The authors, WILEY‐VCH. g) Fluorescence images of HUVECs on the BCS and silicone sensor for 48 h. Reproduced with permission.^[^ ^390^ ^]^ Copyright 2020, The authors, WILEY‐VCH.

Piezoresistive sensors, which can convert pressure or strain into resistance signals, have been used in various industrial applications, especially in wearable devices.^[^ ^388^ ^]^ For example, they can be installed on clothing to monitor human body motions and even subtle motions. Based on these properties, materials used for piezoresistive sensors should have several characteristics, such as light weight, superb flexibility, high sensitivity, electric conductivity, and great biocompatibility. Among all types of BCMs, biomass‐derived carbon aerogels (BCAs) have attracted extensive attention for application in piezoresistive sensors due to their ultralightness and resilience. However, due to the random microstructure of BCAs, their strength and resilience are unsatisfactory and lack linear sensitivity. Therefore, the rational structure design of BCAs is significant for the fabrication of high‐performance piezoresistive sensors. Jiang et al. fabricated carbon aerogels with ordered lamellar structures that were lightweight (8.16 mg cm^−3^) from graphene oxide and water‐soluble cellulose.^[^ ^389^ ^]^ The optimized sample C‐HPMC/rGO‐6 exhibits great mechanical performance with high resilience when it undergoes more than 300 cycles under high compression strain (99%), as shown in **Figure** **18**a. Due to the ordered structure of the carbon aerogel, C‐HPMC/rGO‐6 also possesses a superior linear sensitivity of 15.8 kPa^−1^ over a wide pressure range of 0–18 kPa (Figure 18b). The superb sensitivity can be ascribed to the continuous and ordered morphology of C‐HPMC/rGO‐6 compared with other samples in this work. For incontinuous and disordered samples such as C‐HPMC/rGO‐8, many defects exist in the fragments. When pressure compressive stress is applied, the stress concentration and inefficient transfer of stress will occur in defects. The structure can be collapsed during high strain or cyclic compression. However, the ordered microstructure is beneficial not only to the efficient stress transfer but also to the structure maintenance at high compressive strain. The morphology of C‐HPMC/rGO‐8 and C‐HPMC/rGO‐6 and their corresponding elastic mechanisms are illustrated in Figure 18c.

Inspired by the extraordinary compressibility and superb mechanical strength of *Thalia dealbata* stems, Chen et al. prepared a carbon piezoresistive sensor derived from wood biomass with a novel “bridge support lamellar” structure, which is similar to that of *T. dealbata* stems.^[^ ^390^ ^]^ This structure is able to guarantee the even spreading of the stress and avoid structural deformations when strain is applied, leading to the high compressibility and strength of the sensors, as shown in Figure 18d,e. The piezoresistive sensor can be installed on human fingers to detect finger motion from straight (I) to curved (II), as presented in Figure 18f. In addition, the as‐prepared bioinspired architecture carbonaceous nanofibrous sponge (BCS) is demonstrated to be biocompatible. Figure 18g depicts the HUVECs cultured on BCSs and silicone sensors after 48 h. The stronger green fluorescence on BCS sensors suggests its low cytotoxicity, showing the promising application in clothing or human body implanted sensors.

##### Biomass‐Derived Carbon Materials as Chemical Sensors and Biosensors

**Figure 19:**
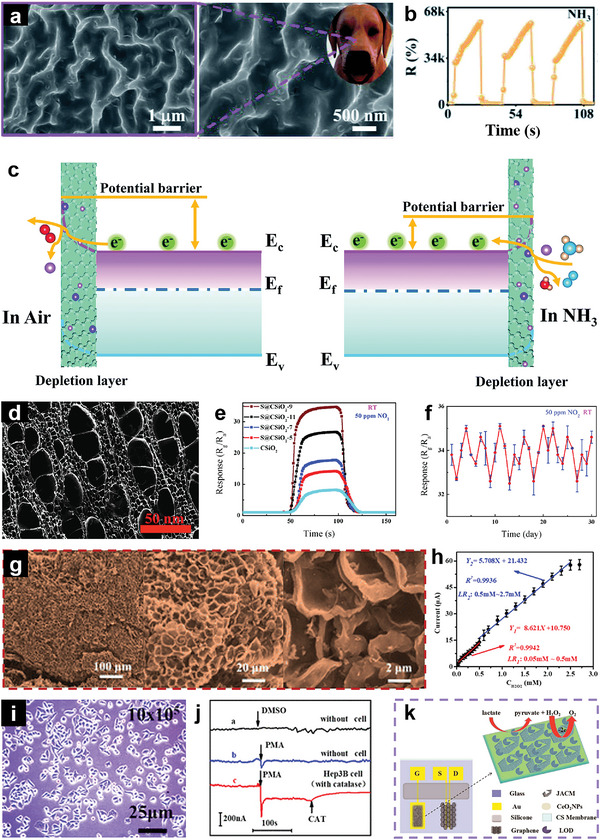
a) SEM images with different resolutions of CRT. Reproduced with permission.^[^ ^392^ ^]^ Copyright 2022, RSC. b) Sensing curves of the CRT toward 500 ppm NH_3_. Reproduced with permission.^[^ ^392^ ^]^ Copyright 2022, RSC. c) NH_3_ detection mechanism of the CRT sensor. Reproduced with permission.^[^ ^392^ ^]^ Copyright 2022, RSC. d) Cross‐sectional SEM image of S@CSiO_2._ Reproduced with permission.^[^ ^393^ ^]^ Copyright 2019, Elsevier. e) Gas response curves of the S@CSiO_2_ samples with various fabrication parameters for 50 ppm NO_2_ at RT. Reproduced with permission.^[^ ^393^ ^]^ Copyright 2019, Elsevier. f) Long‐term gas response stability of the S@CSiO2‐9 device measured with 50 ppm NO_2_ for 30 days. Reproduced with permission.^[^ ^393^ ^]^ Copyright 2019, Elsevier. g) SEM images of TS‐HTC. Reproduced with permission.^[^ ^395^ ^]^ Copyright 2021, ACS. h) Response current of TS‐HTC as a function of H_2_O_2_ concentration. Reproduced with permission.^[^ ^395^ ^]^ Copyright 2021, ACS. i) Microscopic photos of Hep 3B cells. Reproduced with permission.^[^ ^395^ ^]^ Copyright 2021, ACS. j) Real‐time monitoring of H_2_O_2_ released by TS‐HTC with the addition of dimethyl sulfoxide (DMSO) without cultured Hep 3B cells (curve a), the addition of PMA (curve b), and PMA with catalase (curve c) with cultured Hep 3B cells in PBS at −0.3 V (vs Ag/AgCl). Reproduced with permission.^[^ ^395^ ^]^ Copyright 2021, ACS. k) Schematic diagram of a solution‐gated graphene transistor. Reproduced with permission.^[^ ^395^ ^]^ Copyright 2021, ACS.

With the rapid growth of the chemical and engineering industry, gas detection, especially toxic gas detection, is essential for the safety of employees. The development of chemical sensors for gas adsorption and detection has become a research hotspot worldwide. Biomass‐derived porous carbon materials have been demonstrated to be promising materials for chemical sensors.^[^ ^391^ ^]^ Inspired by the pleated structure of dog's maxillary turbinate, Sun et al. fabricated porous carbon materials from rose tea (CRT) as chemical sensors for NH_3_ gas detection.^[^ ^392^ ^]^ The SEM images of CRT with different resolutions in **Figure** **19**a suggest that CRT has a pleated nanostructure similar to the internal rugae structure of the dog nose. CRT shows a high response (60.16 k%) to NH_3_ gas of 500 ppm (Figure 19b), and the theoretical limit of detection is calculated to be 4.82 ppb. The authors also find that the high performance of CRT is contributed by both the biomimetic dog nose structure and the K element in CRT. According to density functional theory calculations, K‐doped CRT has a larger energy of the adsorbed gas than undoped graphene, which proves that the sensitivity enhancement to NH_3_ is achieved by the doped K elements. In addition, the detection mechanism of NH_3_ is proposed in this work, as shown in Figure 19c. The resistance of sensing materials is exponentially related to the effective potential barrier. That is, a slight change in it will lead to a huge change in sensing resistance. When CRT is exposed in air, oxygen can be absorbed on the CRT surface via the reaction of

(6) $$O_{2\left(\mathrm{ads}\right)}\;+{\;e}^{-}\;\to {\;O}_{2}^{-}$$

resulting in the formation of a depletion layer on the surface of CRT. When CRT is exposed to NH_3_, two reactions will occur:

(7) $$2{\mathrm{NH}}_{3}\;+\;\frac{3}{2}O_{2\left(\mathrm{ads}\right)}^{-}\;\to {\;N}_{2}\;+\;3H_{2}O\;+3e^{-}$$

and

(8) $$2{\mathrm{NH}}_{3}\;+\;2O_{2\left(\mathrm{ads}\right)}^{-}\;\to {\;N}_{2}O\;+\;3H_{2}O\;+2e^{-}$$

Thus, the captured electrons are released back into the conduction band of CRT, causing a decrease in the effective potential barrier and the depletion layer of electrons. Wang et al synthesized S‐doped SiO_2_ hybrid carbon materials derived from bamboo leaves (S@CSiO_2_) for NO_2_ sensing.^[^ ^393^ ^]^ The as‐prepared sensor exhibits a 3D mesoporous structure, as presented in Figure 19d. Such structure and S doping endow the carbon material with a high detection sensitivity toward NO_2_ (34.65 for 50 ppm, as shown in Figure 19e) and a detection limit of 0.5 ppm. In addition, the sensing material is highly stable, with a maximum fluctuation of 2.1% over 30 days at RT (Figure 19f).

Due to the chemical stability, strong cytocompatibility and light weight properties of BCMs, they have been employed in biosensors to detect various virus strains or concentrations of various biomacromolecules, such as glucose. Shan et al. prepared a glucose biosensor based on glucose oxidase (GOD)‐anchored 3D porous cane vine stem‐derived carbon.^[^ ^394^ ^]^ The 3D porous structure of the carbon matrix offers more implantation sites for the immobilization of GOD, leading to a high sensitivity of 8.617 µA mm ^−1^ and quite a low LoD of 0.19 µM. Guo et al developed a biosensor based on skin tissue of tamarind seedcase‐derived carbon materials (TS‐HTC) for sensing hydrogen peroxide (H_2_O_2_), a type of reactive oxygen species, which can cause cytopathic effects by invading other cellular compartments.^[^ ^395^ ^]^ TS‐HTC exhibits a highly uniform and ordered porous structure with an SSA of 436.425 m^2^ g^−1^, as shown in Figure 19g. TS‐HTC has an outstanding sensitivity of 122.02 µA mm ^−1^ in the H_2_O_2_ concentration range of 0.05–0.5 mm (Figure 19h). The TS‐HTC‐based biosensor also displays a fast detection response with a short response time of 3.61 s. A real‐time monitoring experiment of the as‐prepared sensor in cells for the detection of liver cancer was carried out. Figure 19i is a microscopic photo of Hep 3B cells, which can emit H_2_O_2_ immediately upon exposure to polymethyl acrylate (PMA). Once PMA is added, the sensor can respond, and a signal peak can be observed (curve c in Figure 19j), suggesting the potential of TS‐HTC for the early diagnosis of diseases. Bi et al. developed a one‐step method to fabricate Jerusalem artichoke stalk‐derived porous carbon‐supported nanoceria for functionalization of solution‐gated graphene transistors as biosensors for real‐time detection of lactic acid from cancer cell metabolism.^[^ ^396^ ^]^ The assembled graphene transistor is illustrated in Figure 19k, where D, S, and G represent the drain, source and gate electrodes, respectively. The biosensor exhibits high lactic acid detection sensitivity with a linear range of 3–300 µM and a low detection limit of 300 nM.

Coronavirus disease‐2019 (COVID‐19) has been a worldwide pandemic since its outbreak in December 2019. Numerous studies have been conducted on the development of vaccines or medicines against COVID‐19.^[^ ^397^ ^]^ As mentioned above, BCMs have huge potential in the field of biosensors. However, researches on BCM‐based biosensors for detecting COVID‐19 have not yet been reported. Corresponding work will be meaningful for the early detection and diagnosis of COVID‐19 to stop its wide spread or outbreak in a region.

#### 5.2.2 Applications in Other Electronics

**Figure 20:**
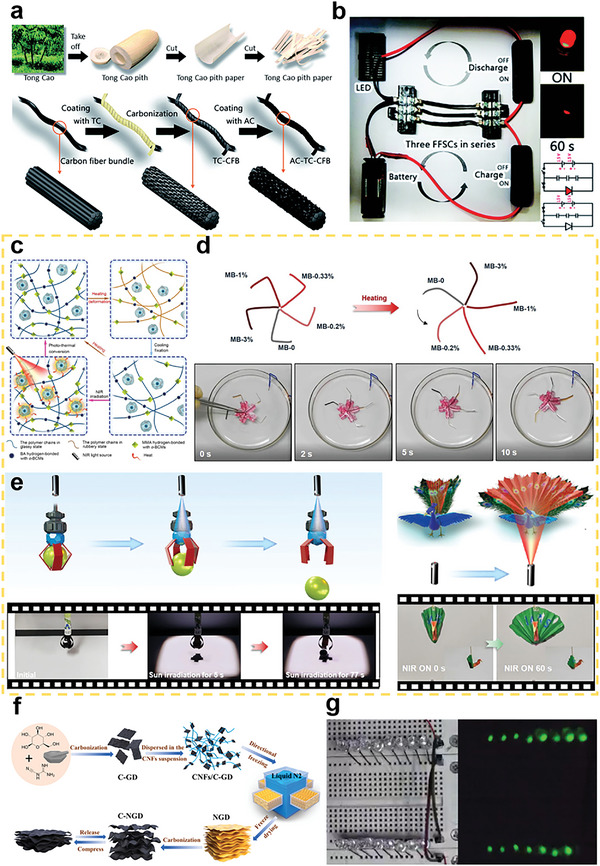
a) Preparation process of the FFSCs. Reproduced with permission.^[^ ^399^ ^]^ Copyright 2020, The authors, RSC. b) Brightness variation of the LED powered by three charged FFSCs arranged in series. Reproduced with permission.^[^ ^399^ ^]^ Copyright 2020, The authors, RSC. c) Shape recovery mechanism of *o*‐BCM‐doped photoresponsive polymer composites. Reproduced with permission.^[^ ^401^ ^]^ Copyright 2022, Springer Nature. d) Schematic diagram and photographs of SMPs with various addition ratios of *o*‐BCMs exposed to warm water at 39 °C. Reproduced with permission.^[^ ^401^ ^]^ Copyright 2022, Springer Nature. e) Diagram and photographs of *o*‐BCM‐doped SMPs assembled into releasing claws and displaying peacock. Reproduced with permission.^[^ ^401^ ^]^ Copyright 2022, Springer Nature. f) Illustration of the preparation process of C‐NGD. Reproduced with permission.^[^ ^402^ ^]^ Copyright 2021, Elsevier. g) Photographs of the assembled TENG device lighting up 15 diode lights. Reproduced with permission.^[^ ^402^ ^]^ Copyright 2021, Elsevier.

With the rapid development of wearable technology, textiles integrated with electronics, also known as smart textiles, have attracted much attention. This kind of fabric can detect external stimuli and convert the stimuli into electric signals or is even able to respond to the stimuli.^[^ ^398^ ^]^ To achieve these functions, the main component of smart textiles is conductive fibers, yarns, or fabrics. Additionally, different from other wearable devices, smart textiles face more mechanical stresses, or environmental corrosion and should be washable. With high electrical conductivity, low toxicity, and great sustainability, BCMs have large potential in smart textile applications. For example, Hsiao et al. took advantage of the honey bomb‐like structure of *Tetrapanax papyrifer* (Tong Cao, TC) to prepare flexible fibrous supercapacitors (FFSCs) in smart textiles. TC can be easily cut into paper and is used to wrap carbon fiber bundles. The composite yarn is further carbonized and coated with commercial activated carbon.^[^ ^399^ ^]^ The preparation process is illustrated in **Figure** **20**a. The as‐fabricated FFSCs in this work have a maximum length energy density of 3.98 µW h cm^−1^ (16.1 µW h cm^−2^) at a power density of 0.07 mW cm^−1^ (1.99 mW cm^−2^). The stability of FFSCs is excellent, with a capacitance retention of 91% over 10 000 cycles. In addition, three FFSCs with a length of 3 cm are assembled and charged for 10 s. The assembled FFSCs are able to power a red LED for more than 60 s, as presented in Figure 20b.

Due to their superb light absorption property, carbon nanomaterials have been employed in photoresponsive shape memory polymer (SMP) composites.^[^ ^400^ ^]^ BCMs are considered to be alternative carbon precursors for this use. BCMs should be designed with excellent dispersibility and great photothermal properties for photothermal conversion agents. In recent work, Yan et al. used oxidized corn straw‐derived carbon materials (*o*‐BCMs) as photothermal conversion agents doped into SMP composites composed of n‐butyl acrylate (BA) and methyl methacrylate.^[^ ^401^ ^]^ With the addition of *o*‐BCMs, the response triggering temperature is decreased to a quite safe one that is below the normal body temperature. In addition, the mechanical performance of SMP composites is enhanced due to the existence of *o*‐BCMs. The photoresponsive mechanism of *o*‐BCMs‐doped polymer composites is illustrated in Figure 20c. *o*‐BCMs can absorb energy from near‐infrared or solar irradiation and convert light energy into heat. When the temperature increases due to the heat, the polymer will be in a rubbery state, and the stored strain energy will be released, thus realizing the shape recovery process. In addition, the shape recovery behavior of polymer composites is related to the *o*‐BCM addition ratio (Figure 20d), and the doping of 1% carbon nanomaterials (MB‐1%) displays superior performance. MB‐1% was also assembled to imitate the releasing claws and the displaying peacock (Figure 20e). The results demonstrate the potential of BCM‐doped photoresponsive SMP composites in smart textiles or intelligent robots.

Long et al. used nitrogen‐doped carbon aerogel from CNFs, glucose, and dicyandiamide (C‐NGD) for multifunctional electronic devices, and the prepared carbon aerogel exhibited a super stable wave‐layered structure, as depicted in Figure 20f.^[^ ^402^ ^]^ Apart from excellent behaviors as wearable pressure sensors and supercapacitors, C‐NGD also exhibits potential application in triboelectric nanogenerators (TENGs). When pressure is applied to the C‐NGD‐based TENG, the electrical balance will be broken, leading to electron flow through the external load, and a new balance will be formed via electron transfer. The assembled TENG can light up 15 diode lights in the test exhibited in Figure 20g. The short‐circuit current (*I*_sc_), open‐circuit voltage (*V*_oc_), and short‐circuit transfer charge (*Q*_sc_) of the TENG device in this work are 3 µA, 38 V, and 12 nC, respectively, demonstrating great performance as TENG devices.

### 5.3 Environmental and Other Applications of Biomass‐Derived Carbon Materials

#### 5.3.1 Biomass‐Derived Carbon Materials for Electromagnetic Interference Shielding

**Figure 21:**
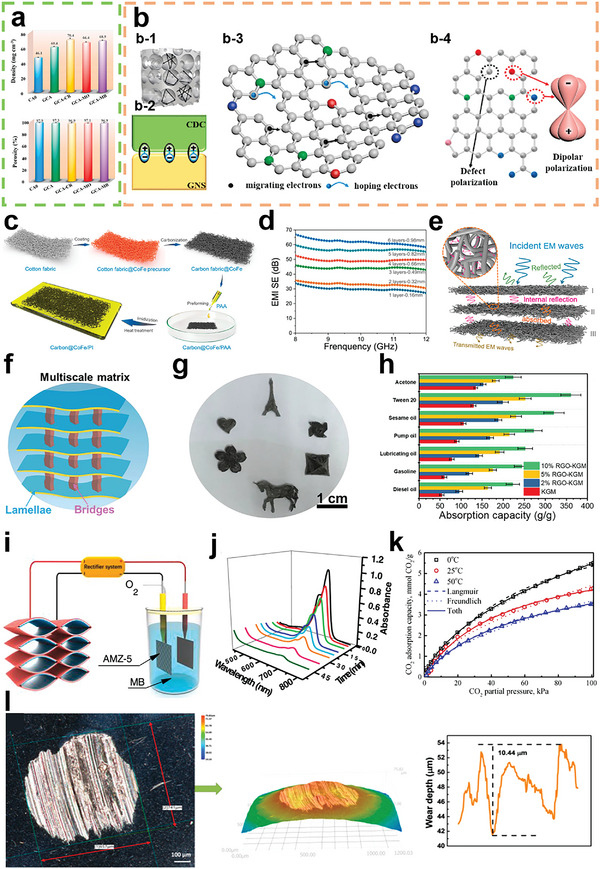
a) Density and porosity of heteroatom doped carbon aerogel derived from graphene oxide nanosheet (GONS)/cellulose composite. Reproduced with permission.^[^ ^407^ ^]^ Copyright 2021, Elsevier. b) Schematic illustration of b‐1) multiple reflection model, b‐2) interfacial polarization in the carbon matrix of the carbon aerogel composites, b‐3) conduction loss, interface polarization, and b‐4) dipole polarization, where the grey, red, blue, green, and purple atoms represent carbon, sulfur, pyridine N, graphite N, and pyrrole N, respectively. Reproduced with permission.^[^ ^407^ ^]^ Copyright 2021, Elsevier. c) Preparation process of nanocomposite CCF@CoFe/PI film. Reproduced with permission.^[^ ^411^ ^]^ Copyright 2020, Elsevier. d) EMI shielding efficiency of nanocomposite CCF@CoFe/PI film with 1–6 layers. Reproduced with permission.^[^ ^411^ ^]^ Copyright 2020, Elsevier. e) Multiple layers EMI shielding mechanism model of nanocomposite CCF@CoFe/PI film. Reproduced with permission.^[^ ^411^ ^]^ Copyright 2020, Elsevier. f) Schematic of the RGO‐KGM carbon aerogel. Reproduced with permission.^[^ ^418^ ^]^ Copyright 2020, ACS. g) Optical photographs of RGO‐KGM carbon aerogel with different shapes. Reproduced with permission.^[^ ^418^ ^]^ Copyright 2020, ACS. h) Absorption capacity of KGM and RGO‐KGM carbon aerogels for various oils and organic liquids in the oil system. Reproduced with permission.^[^ ^418^ ^]^ Copyright 2020, ACS. i) Schematic diagram of the PFW‐TENG based self‐powered EF degradation MB system. Reproduced with permission.^[^ ^422^ ^]^ Copyright 2021, Elsevier. j) UV–vis spectra of MB in the acidic medium EF during degradation. Reproduced with permission.^[^ ^422^ ^]^ Copyright 2021, Elsevier. k) CO_2_ adsorption isotherms of CAC‐S at 0, 25, and 50 °C fitted to different kinetic equations. Reproduced with permission.^[^ ^426^ ^]^ Copyright 2019, Elsevier. l) Optical photographs of worn surfaces and the 3D profile of the wear track of the steel ball after friction tests (at 392 N, 155 °C, 0.3 wt%) of g‐C/Fe_3_O_4_. Reproduced with permission.^[^ ^429^ ^]^ Copyright 2022, ACS.

With the tremendous development of electronic devices for both civil and military utilization, electromagnetic radiation and electromagnetic interference (EMI) emitted by these devices have raised concerns about their threat to human health as well as the operation of communication systems.^[^ ^403^ ^]^ In this case, EMI shielding materials (ESMs) are exploited as protectors to isolate electronics from the surrounding EMI and have significant applications as microwave absorbing materials for military weapons and equipment.^[^ ^404^ ^]^ EMI shielding can be realized by three mechanisms: Reflection loss, adsorption loss, and multiple reflections.^[^ ^405^ ^]^ In addition, hierarchically porous, multilayer, and core–shell structures have been demonstrated to be a design principle for ESMs with high absorption coefficients.^[^ ^406^ ^]^ Based on this strategy, Wang et al. prepared heteroatom‐doped carbon aerogels derived from graphene oxide nanosheet/cellulose composite hydrogels as ESMs.^[^ ^407^ ^]^ It is noteworthy that the heteroatom sources in this work are organic dyes: methylene blue (MB), Congo red (CR), and methyl orange (MO). The as‐prepared carbon aerogels have low density and high porosity (**Figure** **21**a). The optimized sample GCA‐MB shows a superb EMI shielding effectiveness (SE) of 97.3 dB in the X band as well as a high absorption coefficient of 0.69 at a density of 68.9 mg cm^−3^. These performances can be attributed not only to the 3D architecture of the carbon aerogel, which leads to multiple reflections but also to the interfacial polarization, conduction loss and dipole polarization induced by heteroatom doping, as illustrated in Figure 21b.

A double conductive network can produce interfacial polarization and multiple scattering modes of EMWs inside the material, which can enhance dielectric loss and prolong the pathway of EMWs, thus consuming electromagnetic energy by heat.^[^ ^408^ ^]^ Zhou et al. designed a hierarchically porous carbon aerogel derived from cotton linters hybrid with CNTs for the elimination of the secondary pollution of reflected EMW.^[^ ^409^ ^]^ Due to the heterogeneous network of CNT‐ and cotton liner‐derived porous carbon, the resultant sample a‐CCA5.0 exhibits an excellent EMI shielding performance of 96.4 dB and a great absorption coefficient of 0.69 at a density of 68.9 mg cm^−3^. In addition, the surface property of the material is demonstrated to be hydrophobic with a water contact angle of 158.3° and a small slide angle (SA) of 5.3°.

Another effective strategy to enhance EMI shielding performance is to dope BCMs with magnetic or electromagnetic components such as Fe, Co, Ni, to attenuate the incident EMWs by magnetic losses.^[^ ^410^ ^]^ For instance, Li et al. fabricated CoFe nanoparticle‐coated cotton fabric‐derived flexible carbon for EMI shielding.^[^ ^411^ ^]^ The carbonized samples were further encapsulated by polyimide (PI) resin to obtain carbon composite films. Figure 21c depicts the preparation process. Due to the encapsulation of PI, the composite film is flexible and has an excellent tensile strength of 10 MPa. The monolayer composite film possesses a superb EMI shielding property of 32 dB over the X‐band with a thickness of only 0.16 mm. When 6 layers were stacked together, the EMI shielding reached 62 dB over the X band (Figure 21d). The mechanism of multilayer EMI shielding was studied as illustrated in Figure 21e. The electronic microwaves (EMs) encounter the next composite film layer after passing through the first layer; thus, the absorption and reflection processes are repeated. Through the repeated interlayer dissipation and absorption or repeated dielectric loss and magnetic loss process, the incident EMs will be attenuated to a vast degree.

#### 5.3.2 Biomass‐Derived Carbon Materials for Water Treatment

During recent decades, explosive growth in industrial production has created an economic miracle propelling our society forward. However, this miracle comes with a cost to the environment, as industrial production emits pollution at the same time. Waterways worldwide are polluted by toxic heavy metal ions, organic dyes, etc., from industrial effluents.^[^ ^412^ ^]^ Various efforts have been devoted to tackling this issue, and treatment approaches, such as membrane filtration, electrochemical treatment, and absorption, have been developed and adopted. Porous carbon materials derived from biomass with a large SSA and pore volume, tunable surface functionality, mechanical integrity, chemical and thermal stability have been deemed promising materials for water treatment.^[^ ^413^ ^]^ The adsorption performance of porous carbon materials is related to both their porous structure and surface chemistry. Micropores, the size of which is close to the molecular size of organic pollutants, have a high adsorption capacity, while meso‐ and macropores offer a pathway for organics into the inner carbon surfaces.^[^ ^414^ ^]^ Carbon surfaces with *π* electrons are able to form *π*–*π* interactions with organic pollutants containing benzene rings or C = C bonds, indicating that a high level of graphitization is beneficial to adsorption performance.^[^ ^415^ ^]^ In addition, carbon surface functionalities such as N and O can develop hydrogen bond or Lewis acid–base interactions with organics possessing —OH or —COOH functional groups.^[^ ^416^ ^]^

Tian et al. reported the preparation of chemically activated porous carbon materials derived from bottlebrush flowers for binary micropollutant removal in water.^[^ ^417^ ^]^ The activated carbon exhibits a well‐developed porous morphology, and the SSA of the as‐prepared carbon can reach as high as 2025 m^2^ g^−1^. The optimized sample Ni‐NPC‐1 shows the highest adsorption capacity of 91.4 mg g^−1^ and efficient catalysis in phenol and hydroxybenzoic acid degradation with activities of 0.62 and 0.053 min^−1,^ respectively. These performances can be attributed to the large SSA, suitable N content and trace Ni doped into the carbon matrix. Chen et al. designed a 3D reduced graphene oxide‐konjac glucomannan (RGO‐KGM) carbon aerogel with layered and bridged pillared structures (as illustrated in Figure 21f) for oil–water separation.^[^ ^418^ ^]^ This 3D morphology offers a large SSA of 1132 m^2^ g^−1^. The final products can be manufactured into various shapes (Figure 21g) with light weight, superb elasticity, and hydrophobicity. The as‐prepared carbon aerogel absorbent has a high absorption capacity toward different kinds of organic solvents and oils, and the capacity can be as high as 360 g g^−1^, as shown in Figure 21h.

Electron‐Fenton (EF) is another widely used water treatment technology that works on the oxidation of organic pollutants through electrochemical processes.^[^ ^419^ ^]^ Organic pollutants are oxidized by the in situ electrogenerated H_2_O_2_ during the two‐electron ORR and hydroxyl radicals (•OH) with strong oxidation in the presence of Fe^2+^.^[^ ^420^ ^]^ BCMs with a large SSA and excellent conductivity have been used as cathodes for the EF process. Ganiyu et al. fabricated carbon foam derived from sucrose with an SSA of 160 m^2^ g^−1^ for EF degradation of sulfanilamide.^[^ ^421^ ^]^ With the carbon foam cathode, the in situ production of H_2_O_2_ is up to 7 mg L^−1^ at 60 mA, and it also contributes to the complete degradation and chemical oxygen demand removal of 0.5 mm synthetic sulfanilamide solution in less than 4 h of treatment. The doping of N‐ and oxygen‐containing functional groups into a carbon skeleton is one of the strategies used to prepare EF cathodes. Zhu et al. prepared N‐doped porous carbon materials from Artemisia argyi as cathodes for EF degradation toward methylene blue (MB).^[^ ^422^ ^]^ The porous carbon cathode has a high N content of 4.0 at% and a large SSA of 1302 m^2^ g^−1^. It is noteworthy that they fabricated a printed flexible waved triboelectric nanogenerator using 3D printing technology to power the EF system. The schematic diagram is presented in Figure 21i. During the EF process, the decrease in the MB characteristic absorption peaks at 664 nm with increasing reaction time can be observed by ultraviolet–visible (UV–vis) spectroscopy (Figure 21j). The MB degradation efficiency reached 98.1% within 58 min, and the degradation efficiency was positively related to the content of C—O—C and —COOH in the carbon materials.

#### 5.3.3 Other Applications

The continuously increasing level of carbon dioxide in the atmosphere, also known as the greenhouse gas, which has been considered the major factor of climate change, has raised increasing concern. One of the prevalent solutions is to capture and store CO_2_ and thus decrease the CO_2_ concentration in the atmosphere.^[^ ^423^ ^]^ BCMs with high SSA have been applied as solid absorbents of CO_2_. In addition, the porous structure should be designed with micropore sizes smaller than 0.7–0.9 nm, which have been demonstrated for better CO_2_ adsorption performance.^[^ ^424^, ^425^ ^]^ Guo et al. reported the preparation of sugarcane bagasse‐derived porous carbon materials by chemical or physical activation for CO_2_ capture.^[^ ^426^ ^]^ The carbon activated by NaOH (CAC‐S) has the largest SSA of 1149 m^2^ g^−1^. It is reported that the isothermal CO_2_ uptakes of CAC‐S are the highest among all the samples in this work, with values of 5.50 and 4.28 mmol CO_2_ g^−1^ at 0 °C and 25 °C and 1 bar, respectively (Figure 21k). Ma et al. fabricated activated carbon derived from tobacco stems with an ultrahigh SSA of 3839 m^2^ g^−1^ for CO_2_ capture.^[^ ^427^ ^]^ Due to the highly porous structure, the CO_2_ uptake of the materials is 29.5 mmol g^−1^ at 30 bar and 25 °C.

Friction and wear can affect the service life of machines and increase energy consumption. Therefore, developing anti‐friction and anti‐wear materials, such as lubricant additives, has attracted much attention. Although metal nanoparticles such as Fe_3_O_4_ exhibit great anti‐friction properties and facile pretreatment, they often face the problems of poor dispersion and easy aggregation.^[^ ^428^ ^]^ To solve these problems, Chen et al. fabricated graphitized C/Fe3O4 magnetic nanocomposites (g‐C/Fe_3_O_4_) with interpenetrating network structures using cassava starch as the carbon source via hydrothermal and ball milling.^[^ ^429^ ^]^ This nanocomposite is used as a lubricant additive in rapeseed oil and shows superb tribological properties with a high extreme pressure of 804 N and a low friction coefficient of 0.029 at 155 °C. In addition, the wear scar depth of g‐C/Fe_3_O_4_ is the smallest among all the samples, which is 10.44 µm according to the 3D profile of the wear scar in Figure 21l.

## 6 Conclusions and Future Perspectives

**Scheme 2:**
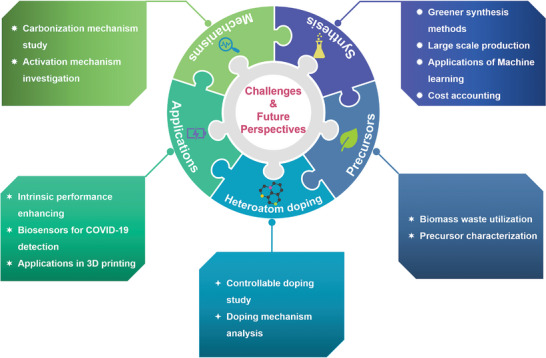
A summary of challenges and outlooks with regard to the mechanism study, synthesis methods, biomass precursors, research on heteroatom doping, and the applications of BCMs.

In summary, different types of biomass precursors and structures of BCMs are introduced in this review. The representative synthetic methods and their features are discussed in detail. In addition, recent progress of BCMs in versatile applications is reviewed. These summaries and analysis demonstrate that significant advances have been achieved in the research of BCMs and this field of research continues to be of great interest. Converting biomass into functional carbon materials has high economic value and is conducive to sustainable development. Nevertheless, despite recent achievements in the research of BCMs, many challenges remain to be addressed. These remaining challenges and several future perspectives are summarized in **Scheme** **2** and then described in detail in this section.

### 6.1 Biomass Precursors

More attention should be given to the reutilization of biomass waste as a BCM precursor. As mentioned in this review, a large amount of biomass waste is produced annually, and reutilization can be a sustainable and economical way to process biomass waste. At present, the characterizations of biomass precursors, which are closely related to the structures of the obtained BCMs, are notably insufficient in the reported literature. For instance, heteroatom‐doped BCMs are widely reported, while the content of heteroatoms in biomass precursors has rarely been characterized and reported in the literature. In addition, because of the changeable compositions of the biomass precursor, the structure of the carbon materials produced from the same type of biomass precursor may vary vastly. Therefore, pretreatments of the selected precursor, including chemical purification or extraction, are beneficial to achieve the controllable synthesis of BCMs.

### 6.2 Synthesis of Biomass‐Derived Carbon Materials

To date, traditional synthesis methods, such as pyrolysis and HTC, are well developed to convert biomass into carbonaceous materials. However, many of these approaches are time‐ and energy‐consuming. Novel carbonization methods, including laser‐induced carbonization and microwave‐assisted carbonization, have been exploited recently. However, the carbonization process of these methods still occurs at high temperatures. Therefore, research on low‐temperature graphitization would be worthwhile considering their low energy consumption. Greener synthesis methods with less pollutant emission and less energy consumption are desirable.

Another challenge for the synthesis of BCMs is to realize large‐scale production. In the reported research work, the maximum production reported is on the gram scale and cannot meet the requirements for industrialization or commercialization. Meanwhile, machine learning methods should be further developed for the rational design and precise control of BCMs synthesis. At present, the experimental data base is not sufficient for the construction of a valid machine learning model. More collaborations between materials scientists and experts in machine learning are necessary to achieve this goal. Moreover, although the cost‐effectiveness of BCMs has been addressed in some studies, few studies have incorporated cost accounting.

### 6.3 Study on Formation Mechanisms

Pyrolysis of biomass is the most critical process of BCM fabrication. Numerous formation mechanisms of BCM during carbonization have been proposed. However, these mechanisms, which are mainly based on the analysis of gaseous products and mass loss during carbonization, cannot sufficiently determine the sequence of reactions that occur and the exact transformation stages. Therefore, in situ characterizations are urgently required to probe the detailed formation mechanisms, which are essential to achieve the controllable synthesis of BCMs. In addition, in situ technologies are expected to probe the mechanism of chemical activation during carbonization for the preparation of porous BCMs with ultrahigh SSA and porosity.

### 6.4 Heteroatom Doping

Many studies have demonstrated that heteroatom‐doped carbon materials exhibit better electrochemical performance because heteroatom doping will increase defects and active sites and modify the surface properties. However, controllable heteroatom doping remains a challenge for BCM synthesis. The heteroatom doping content is often unpredictable, and the doping mechanism is still unclear. For example, doped N atoms exist as various types, including pyrrolic N, pyridinic N, graphitic N, and oxidized N, but the exact role of each N type in enhancing BCM performance still needs further research. Moreover, studies on the relation between carbonization parameters and heteroatom doping are urgently needed for tunable heteroatom doping.

### 6.5 Applications of Biomass‐Derived Carbon Materials

For some applications, the intrinsic performance of BCMs is unsatisfactory. For instance, BCMs lack active sites as electrocatalysts and often serve as supports for metal catalysts. The addition of metal not only increases the cost but also involves the use of chemical reagents. Therefore, future research should focus on determining the exact active sites of BCMs via in situ characterizations or theoretical calculations. This can provide guidance for fabricating BCMs with rich active sites, which can enhance the intrinsic performance of BCMs and reduces the load mass of metal catalysts. Besides, there are possible new applications for BCMs. The first one is BCM‐based biosensors for detecting COVID‐19. The outbreak of COVID‐19 has caused significant global morbidity and mortality and has restrained the development of global economy. Although BCMs have shown huge potential in the field of biosensors, no research on BCM‐based biosensors for detecting COVID‐19 has yet been reported. Related work will be meaningful to achieve the timely diagnosis of COVID‐19 to stop its further spread in a region. Another possible application for BCMs is in 3D printing. In recent years, the fabrication of materials with complex geometric shapes in a fast and low‐cost way has been realized due to the development of 3D printing technology. Carbon materials have been widely used in 3D printing as the conductive additives for printable inks. However, the use of BCMs in 3D printing has not yet been studied. We believe 3D printing technology is able to facilitate the applications of BCMs, especially in energy, electronics, and engineered composite materials.

This review has provided an overview of the most recent progress of preparation methods, properties and applications of BCMs and has proposed current challenges and directions for future development of BCMs. We hope this review can accelerate the future development of BCMs in wide‐ranging new applications.

## Conflict of Interest

The authors declare no conflict of interest.
